# Analysis of Chaotic Dynamics by the Extended Entropic Chaos Degree

**DOI:** 10.3390/e24060827

**Published:** 2022-06-14

**Authors:** Kei Inoue

**Affiliations:** Faculty of Engineering, Sanyo-Onoda City University, 1-1-1 Daigaku-Dori, Sanyo-Onoda, Yamaguchi 756-0884, Japan; kinoue@rs.socu.ac.jp

**Keywords:** chaos, Lyapunov exponent, extended entropic chaos degree

## Abstract

The Lyapunov exponent is the most-well-known measure for quantifying chaos in a dynamical system. However, its computation for any time series without information regarding a dynamical system is challenging because the Jacobian matrix of the map generating the dynamical system is required. The entropic chaos degree measures the chaos of a dynamical system as an information quantity in the framework of Information Dynamics and can be directly computed for any time series even if the dynamical system is unknown. A recent study introduced the extended entropic chaos degree, which attained the same value as the total sum of the Lyapunov exponents under typical chaotic conditions. Moreover, an improved calculation formula for the extended entropic chaos degree was recently proposed to obtain appropriate numerical computation results for multidimensional chaotic maps. This study shows that all Lyapunov exponents of a chaotic map can be estimated to calculate the extended entropic chaos degree and proposes a computational algorithm for the extended entropic chaos degree; furthermore, this computational algorithm was applied to one and two-dimensional chaotic maps. The results indicate that the extended entropic chaos degree may be a viable alternative to the Lyapunov exponent for both one and two-dimensional chaotic dynamics.

## 1. Introduction

The Lyapunov exponent (LE) is the most commonly used measure for quantifying the chaos of non-linear dynamical systems. The LE measures the average exponential separation rate of orbits with infinitesimally close initial points. The orbit produced by a smooth map *f* on $R^{d}$ is referred to as chaotic if the largest LE among all *d* LEs is positive. In principle, the Jacobian matrix: $J_{n}\left(x\right)=Df^{n}\left(x\right)$ is necessary to compute LEs. However, in general, obtaining an explicit formula for $J_{n}\left(x\right)$ for a large *n* is challenging. In actual numerical computations, the LEs of a map *f* are obtained by approximating the image ellipsoid $J_{n}U$ of the unit sphere *U*. This approach involves the chain rule and the Gram–Schmidt orthogonalization procedure to compute the LEs of the map *f* [1]. Subsequently, all the LEs of the map *f* on $R^{d}$ can be computed, provided the Jacobian matrix $J_{1}\left(x\right)=Df\left(x\right)$ can be obtained.

Thus, LEs for a time series are generally incomputable in the absence of any information regarding the Jacobian matrix, $J_{1}\left(x\right)$. Therefore, researchers have suggested various estimation methods of LEs for a time series [2,3,4,5,6,7]. The largest LE for a time series may be estimated using these methods. However, estimating all the LEs and their total sum for the time series is not always possible.

The chaos degree quantifies the chaos of a dynamical system as follows: $C\left(\Lambda^{*}\varphi \right)-T\left(\varphi ;\Lambda^{*}\right)$ in Information Dynamics [8]. Here, φ is referred to as the state and $\Lambda^{*}$ as a channel associated with the state change $\varphi \to \Lambda^{*}\varphi$. C(φ) is the complexity of the state φ and $T(\varphi ;\Lambda^{*})$ is the transmitted complexity associated with the state change $\varphi \to \Lambda^{*}\varphi$. A channel $\Lambda^{*}$ is referred to as chaotic in the definition of Information Dynamics, provided chaos degree is positive. In a classical dynamical system, state φ and channel $\Lambda^{*}$ are provided as a probability distribution $p^{\left(n\right)}$ at time *n* and a transition probability matrix from $p^{\left(n\right)}$ at time *n* to $p^{\left(n+1\right)}$ at time n+1. By substituting the Shannon entropy $S(\Lambda^{*}p^{\left(n\right)})$ and the mutual entropy $I(p^{\left(n\right)};\Lambda^{*})$ for $C(\Lambda^{*}\varphi )$ and $T(\varphi ;\Lambda^{*})$ respectively, the entropic chaos degree (ECD) is obtained from $S\left(\Lambda^{*}p^{\left(n\right)}\right)-I\left(p^{\left(n\right)};\Lambda^{*}\right)$ in classical dynamical systems [9]. Thus, the ECD becomes an information quantity equivalent to conditional entropy: $S\left(p^{\left(n+1\right)}\left|p^{\left(n\right)}\right.\right)$ in classical dynamical systems. The ECD offers the advantage of being directly computable for time-series data, even if the dynamical equation generating the time-series data is unknown. Using the ECD, an attempt to characterize chaotic behaviors has been made [9,10,11].

There exists a relationship between the LE and ECD [12]. Unfortunately, the ECD is not always sufficient to be used as an alternative to the LE because it always attains a higher value than the LE for any chaotic map [13]. Therefore, based on the interpretation of the difference between the ECD and LE, an improved ECD was proposed for a one-dimensional chaotic map, and it was shown that the improved ECD is equivalent to the LE under typical chaotic conditions [13,14]. Furthermore, the extended entropic chaos degree (EECD) was introduced as an extended improved ECD to a multidimensional chaotic map. Further, it has also been shown that the EECD coincides with the sum of all LEs in typical chaotic conditions [15].

However, the above relationship between the EECD and LEs assumes several conditions, such that the numbers of mapping points and all components of the equipartition of *I* in the map from *I* to *I* must take the limit of infinity. However, these numbers must be set as finite numbers in actual numerical computations. Therefore, an improved calculation formula for the EECD was proposed, such that the EECD is almost computable as the sum of all LEs of a typical multidimensional chaotic map in actual numerical computations [16].

This study shows that all LEs of a multidimensional chaotic map can be estimated using an improved calculation formula for the EECD and proposes a computational algorithm for the EECD. Moreover, the computational algorithm of the EECD was applied to specific typical chaotic maps.

## 2. Entropic Chaos Degree

This section briefly reviews the definition of the ECD for a difference equation system.

Let *f* be a map, such that f:I→I ($\equiv {\left[a,b\right]}^{d}$). Consider the following difference equation:

$$x_{n+1}=f\left(x_{n}\right),\;n=0,1,\ldots .$$

Let $x_{0}$ be an initial value and let $\left\{A_{i}\right\}$ be a finite partition of *I* such that:

$$I=\underset{k}{⋃}A_{k},\;\;A_{i}\cap A_{j}=\emptyset \;\left(i\neq j\right),\;\;$$

where $A_{i}$ is a Borel measurable subset of *I*.

Then, the probability distribution $\left(p_{i,A}^{\left(n\right)}\left(M\right)\right)$ at time *n* is expressed as

$$\begin{array}{ccc} p_{i,A}^{\left(n\right)}\left(M\right) & = & \frac{1}{M}\sum_{k=n}^{n+M-1}1_{A_{i}}\left(x_{k}\right)\\ & = & \frac{\left|\left\{x_{k}\in A_{i};\;n\leq k\leq n+M-1\right\}\right|}{M} \end{array}$$

and the joint distribution $\left(p_{i,j,A}^{\left(n,n+1\right)}\left(M\right)\right)$ at times *n* and n+1, associated with the difference equation, is expressed as:

$$\begin{array}{ccc} p_{i,j,A}^{\left(n,n+1\right)}\left(M\right) & = & \frac{1}{M}\sum_{k=n}^{n+M-1}1_{A_{i}}\left(x_{k}\right)1_{A_{j}}\left(x_{k+1}\right)\\ & = & \frac{\left|\left\{\left(x_{k},x_{k+1}\right)\in A_{i}\times A_{j};\;n\leq k\leq n+M-1\right\}\right|}{M}, \end{array}$$

where $1_{A}$ is the characteristic function of the set *A*.

Subsequently, the ECD *D* of an orbit $\left\{x_{n}\right\}$ is defined as in [8] as:

(1) $$\begin{array}{ccc} D^{\left(M,n\right)}\left(A,f\right) & = & \sum_{i}\sum_{j}p_{i,j,A}^{\left(n\right)}\left(M\right)\log\frac{p_{i,A}^{\left(n\right)}\left(M\right)}{p_{i,j,A}^{\left(n,n+1\right)}\left(M\right)}\\ & = & \sum_{i}\sum_{j}p_{i,A}^{\left(n\right)}\left(M\right)\left(-\sum_{j=1}^{N}p_{A}^{\left(n\right)}\left(j|i\right)\left(M\right)\log p_{A}^{\left(n\right)}\left(j|i\right)\left(M\right)\right), \end{array}$$

where

$$p_{A}^{\left(n\right)}\left(j|i\right)\left(M\right)=\frac{p_{i,j,A}^{\left(n,n+1\right)}\left(M\right)}{p_{i,A}^{\left(n\right)}\left(M\right)}$$

is the conditional probability from one component $A_{i}$ to another $A_{j}$ for the finite partition $\left\{A_{i}\right\}$ of *I*.

Further, using the ECD, the orbit $\left\{x_{n}\right\}$ associated with the map *f* is uniquely determined in the definition of Information Dynamics (ID) in [8] as follows:

$$\begin{array}{ccc} D^{\left(M,n\right)}\left(A,f\right)>0 & ⟺ & \mathrm{The}\;\mathrm{orbit}\;\{x_{n}\}\;\mathrm{is}\;\mathrm{chaotic}\;\mathrm{in}\;\mathrm{ID},\\ D^{\left(M,n\right)}\left(A,f\right)=0 & ⟺ & \mathrm{The}\;\mathrm{orbit}\;\{x_{n}\}\;\mathrm{is}\;\mathrm{stable}\;\mathrm{in}\;\mathrm{ID}. \end{array}$$

Here, the ECD is denoted as $D^{\left(M\right)}\left(A,f\right)$ without *n*, provided the orbit $\left\{x_{n}\right\}$ does not depend on time *n*. In a similar manner, the ECD is denoted as $D^{\left(M,n\right)}\left(A\right)$ without *f*, provided the orbit $\left\{x_{n}\right\}$ is not generated by the map *f*.

However, the unique definitions of the orbit in ID may not be consistent with the original properties of the orbit. The basic properties of the ECD in [12] are briefly reviewed.

Let *M* be a sufficiently large natural number and let *f* be a one-dimensional map from *I* to *I* where I=[a,b]. Let $\left\{A_{i}\right\}$ be the *L*-equipartition of *I*, such that

(2) $$I=\overset{L-1}{\underset{i=0}{⋃}}A_{i},$$

where

$$A_{i}=\left\{\begin{array}{cc} \left[a_{+}\frac{b-a}{L}i,\;a+\frac{b-a}{L}\left(i+1\right)\right)\; & (i=0,1,\ldots ,L-2),\\ \; & \\ \left[a+\frac{b-a}{L}\left(L-1\right),\;b\right]\; & \left(i=L-1\right) \end{array}\right.$$

Subsequently, the following theorems are proved in [12]:

**Theorem** **1.** *If the map f creates a stable periodic orbit, then the following equality holds:*

(3) $$D^{\left(M,n\right)}\left(A,f\right)=0$$

*for the L-equipartition $\left\{A_{i}\right\}$ of I=[a,b].*

**Theorem** **2.** *Further, if the LE of f is positive, the following inequality holds:*

(4) $$D^{\left(M,n\right)}\left(A,f\right)>0$$

*for the L-equipartition $\left\{A_{i}\right\}$ of I=[a,b].*

**Theorem** **3.** *Let λ(f),λ(g) be the LEs of f,g such that f,g are differentiable almost everywhere in I. Assume that the absolute values $\left|\frac{df}{dx}\left(x\right)\right|,\left|\frac{dg}{dx}\left(x\right)\right|$ are constants for all x∈I.*

*If λ(f)≥λ(g)>0, the following inequality holds for sufficiently large M:*

(5) $$D^{\left(M,n\right)}\left(A,f\right)>D^{\left(M,n\right)}\left(A,g\right)$$

*for the L-equipartition $\left\{A_{i}\right\}$ of I=[a,b].*

However, in Theorem 2, not vice versa because $D^{\left(M,n\right)}\left(A,f\right)>0$ for a quasi-periodic orbit [12]. In Theorem 3, it is assumed that the maps f,g are piecewise linear functions, such as the Bernoulli shift map and the tent map.

Next, the relationship between the ECD and the metric entropy is focused on. Let *T* be a measurable transformation from *I* to *I*, preserving a probability measure μ on *I*, and ξ provides a measurable partition of *I*. Then, the metric entropy of *T* with respect to μ and ξ of *I* is defined by in [17],

(6) $$h_{\mu }\left(T,\xi \right)=\lim_{n\to \infty }\frac{1}{n}H_{\mu }\left(\xi_{n}\right),\;\xi_{n}=\overset{n-1}{\underset{i=0}{⋁}}T^{-i}\xi .$$

Then, for sufficiently large *M*, ECD $D^{\left(M\right)}\left(\xi ,T\right)$ is equal to or larger than the metric entropy $h_{\mu }\left(T,\xi \right)$: see [16].

Using the ECD, the characterization of certain chaotic behaviors has been attempted by the authors of papers such as [9,10,11]. Unfortunately, the ECD is not always sufficient for use as an alternative to the LE because the ECD always attains a higher value than the LE for chaotic maps [13].

## 3. Extended Entropic Chaos Degree

This section briefly reviews the definition of the EECD for a difference equation system.

Let $\left\{A_{i}\right\}$ be the $L^{d}$-equipartition of $I=\prod_{l=1}^{d}\left[a_{l},b_{l}\right]$, such that

(7) $$\begin{array}{ccc} & & I=\overset{L^{d}-1}{\underset{i=0}{⋃}}A_{i},\;A_{i}=\prod_{k=1}^{d}A_{i_{k}}^{\left(k\right)},\;i_{k}=0,1,\ldots ,L-1, \end{array}$$

where

$$A_{i_{k}}^{\left(k\right)}=\left\{\begin{array}{cc} \left[a_{k}+\frac{b_{k}-a_{k}}{L}i_{k},\;a_{k}+\frac{b_{k}-a_{k}}{L}\left(i_{k}+1\right)\right) & (i_{k}=0,1,\ldots ,L-2),\\ \left[a_{k}+\frac{b_{k}-a_{k}}{L}\left(L-1\right),\;b_{k}\right] & \left(i_{k}=L-1\right) \end{array}\right.$$

for k=1,…,d.

Further, for any component $A_{i}$ of $\left\{A_{i}\right\}$, another component $A_{j}$ is divided into the equipartition ${\left\{B_{l}^{\left(i,j\right)}\right\}}_{0\leq l\leq {\left(S_{i,j}\right)}^{d}-1}$ of smaller components, such that

$$A_{j}=A_{{\left(j_{1}\cdots j_{d}\right)}_{L}}=\overset{{\left(S_{i,j}\right)}^{d}-1}{\underset{l=0}{⋃}}B_{l}^{\left(i,j\right)},\;B_{l}^{\left(i,j\right)}=B_{{\left(l_{1}\cdots l_{d}\right)}_{S_{i,j}}}^{\left(i,j\right)}=\prod_{k=1}^{d}B_{l_{k}}^{\left(i,j,k\right)}$$

where

$$B_{l_{k}}^{\left(i,j,k\right)}=\left\{\begin{array}{cc} \left[{\hat{a}}_{k}+\frac{{\hat{b}}_{k}-{\hat{a}}_{k}}{S_{i,j}}l_{k}+\frac{{\hat{b}}_{k}-{\hat{a}}_{k}}{S_{i,j}}\left(l_{k}+1\right)\right) & \left(l_{k}=0,1,\ldots ,S_{i,j}-2,\;S_{i,j}\geq 2\right)\\ \left[{\hat{a}}_{k}+\frac{{\hat{b}}_{k}-{\hat{a}}_{k}}{S_{i,j}}\left(S_{i,j}-1\right),{\hat{b}}_{k}\right] & \left(l_{k}=S_{i,j}-1\right) \end{array}\right.$$

and

$$\begin{array}{ccc} {\hat{a}}_{k} & = & a_{k}+\frac{b_{k}-a_{k}}{L}i_{k},\\ {\hat{b}}_{k} & = & \left\{\begin{array}{cc} a_{k}+\frac{b_{k}-a_{k}}{L}\left(i_{k}+1\right) & \left(i_{k}=0,1,\ldots ,L-2\right)\\ b_{k} & \left(i_{k}=L-1\right) \end{array}\right. \end{array}$$

for k=1,…,d.

Using the function $g_{i,j}$ for any two components $A_{i},A_{j}\;\left(i\neq j\right)$ of $\left\{A_{i}\right\}$, function $R(S_{i,j})$ is introduced by

$$R\left(S_{i,j}\right)=\frac{\sum_{l=0}^{{\left(S_{i,j}\right)}^{d}-1}g_{i,j}\left(B_{l}^{\left(i,j\right)}\right)}{{\left(S_{i,j}\right)}^{d}}.$$

here, the numerator of $R(S_{i,j})$ is the number of $B_{l}^{\left(i,j\right)}$ in $A_{j}\cap f\left(A_{i}\right)$ for any $A_{i}$ and $A_{j}$ and the denominator of $R(S_{i,j})$ is the number of $B_{l}^{\left(i,j\right)}$ in $A_{j}$ for any $A_{i}$ and $A_{j}$. Thus, $R(S_{i,j})$ represents the volume rate of $A_{j}\cap f\left(A_{i}\right)$ to $A_{j}$ at the $1/S_{i,j}$ scale. Moreover, it was directly obtained from [15]

(8) $$\lim_{S_{i,j}\to \infty }R\left(S_{i,j}\right)=\frac{m(A_{j}\cap f\left(A_{i}\right)}{m(A_{j})},$$

where *m* denotes the Lebesgue measure of $R^{d}$.

Then, the EECD $D_{S}$ is defined in [15] as

$$\begin{array}{ccc} D_{S}^{\left(M,n\right)}\left(A,f\right) & = & \sum_{i=0}^{L^{d}-1}p_{i,A}^{\left(n\right)}\left(M\right)\sum_{j=0}^{L^{d}-1}p_{A}^{\left(n\right)}\left(j|i\right)\left(M\right)\log\frac{R(S_{i,j})}{p_{A}^{\left(n\right)}\left(j|i\right)\left(M\right)}, \end{array}$$

where $S={\left(S_{i,j}\right)}_{0\leq i,j\leq L^{d}-1}$,

Clearly, the EECD becomes the ECD only if $R(S_{i,j})=1$ for any $A_{i}$ and $A_{j}$. In other words, from Equation (8), the ECD always regards $m(A_{j}\cap f\left(A_{i}\right))$ as $m(A_{j})$ in the infinite limit of $S_{i,j}$. This results in a difference between the ECD and LE for chaotic maps [15].

First, the following theorem holds with respect to a periodic orbit [15]:

**Theorem** **4.** *Let L,M be sufficiently large natural numbers. If map f creates a stable periodic orbit with period T, then the following equality holds:*

(9) $$D_{S}^{\left(M,n\right)}\left(A,f\right)=-\frac{d}{T}\sum_{k=1}^{T}\log S_{i_{k},j_{k}}.$$

*where $p_{i_{k},A}^{\left(n\right)}\left(M\right)=\frac{1}{T}$, $f\left(A_{i_{k}}\right)=A_{j_{k}}$$\left(i_{k}\neq j_{k}\right)$, k=1,2,…,T.*

Second, the relationship between the EECD and LE in a chaotic dynamical system is briefly reviewed. Let map *f* be a (piecewise) $C^{1}$ function on $R^{d}$. For any $x={\left(x_{1},x_{2},\ldots ,x_{d}\right)}^{t}$, $y={\left(y_{1},y_{2},\ldots ,y_{d}\right)}^{t}$ $\in A_{i}$, let $\hat{J}$ be an approximate Jacobian matrix, such that

$$\hat{J}\left(x,y\right)={\left(\frac{f_{i}\left(x_{1},\ldots ,y_{j},\ldots ,x_{d}\right)-f_{i}\left(x_{1},\ldots ,x_{j},\ldots ,x_{d}\right)}{y_{j}-x_{j}}\right)}_{1\leq i,j\leq d}.$$

Let $r_{k}\left(x,y\right)$(k=1,2,…,d) be the eigenvalues of $\sqrt{{\hat{J}}^{t}\left(x,y\right)\hat{J}\left(x,y\right)}$.

Now, let us consider a piecewise linear function $\hat{f}$ for a (piecewise) $C^{1}$ function *f* such that:

(10) $$\hat{f}\left(x;L\right)\equiv \sum_{i_{1},\ldots ,i_{d}=0}^{L-1}\hat{f}\left(x;\;i_{1},\ldots ,i_{d}\right)1_{A_{i}}\left(x\right),\;\;i=\sum_{k=0}^{d-1}i_{k}L^{k},\;\;i_{k}\in \left\{0,1,\ldots ,L-1\right\},$$

where

(11) $$\hat{f}\left(x;\;i_{1},\ldots ,i_{d}\right)\equiv \hat{J}\left(\hat{x},\hat{x}+h\right)\left(x-\hat{x}\right)+f\left(\hat{x}\right)$$

Here, $\hat{x}={\left({\hat{x}}_{1},{\hat{x}}_{2},\ldots ,{\hat{x}}_{d}\right)}^{t}$ is randomly sampled from $A_{i}$ and $h={\left(h_{1},h_{2},\ldots ,h_{d}\right)}^{t}$ such that $∥h∥\ll ∥\hat{x}∥$ where ${∥h∥}^{2}=h_{1}^{2}+h_{2}^{2}\cdots +h_{d}^{2}$.

In order to consider the piecewise linear function $\hat{f}$ as an approximate formula of the (piecewise) $C^{1}$ function *f*, the following assumption is introduced.

**Assumption** **1.** *Assume that for sufficiently large natural numbers L and M, the points x in $A_{i}$ are uniformly distributed over $A_{i}$, such that, for any subset $B_{i}$ of $A_{i}$*

(12) $$\lim_{n\to \infty }\frac{1}{n}C\left(x,B_{i},n\right)=\frac{m\left(B_{i}\right)}{m\left(A_{i}\right)}$$

*where m is the Lebesgue measure on $R^{d}$ and $C\left(x,B_{i},n\right)$ is the number of points included in $B_{i}$ among n points, which are randomly sampled from $A_{i}$.*

Then, the following theorem is proven with respect to an aperiodic orbit.

**Theorem** **5.** *Let f be a (piecewise) $C^{1}$ function. Then the following equality is valid.*

$$\lim_{L\to \infty }\lim_{S\to \infty }\lim_{M\to \infty }D_{S}^{\left(M,n\right)}\left(A,f\right)=\sum_{k=1}^{d}\lambda_{k}\left(f\right),$$

*where*

$$S\to \infty \Leftrightarrow S_{i,j}\to \infty \;\left(i,j=0,1,\ldots ,L^{d}-1\right)$$

*and $\left\{\lambda_{1}\left(f\right),\ldots ,\lambda_{d}\left(f\right)\right\}$ represent the Lyapunov spectrum of the map f.*

**Proof.**  Let $\hat{f}$ be a (piecewise) linear function given as Equation (10) for a (piecewise) $C^{1}$ function *f* under Assumption 1.

As shown in Section 4, for a large natural number *L*,

(13) $$\lim_{S\to \infty }\lim_{M\to \infty }D_{S}^{\left(M,n\right)}\left(A,\hat{f}\right)=\sum_{k=1}^{d}\lambda_{k}\left(\hat{f}\right).$$

From Equation (13),

(14) $$\begin{array}{ccc} \lim_{L\to \infty }\lim_{S\to \infty }\lim_{M\to \infty }D_{S}^{\left(M,n\right)}\left(A,f\right) & = & \lim_{L\to \infty }\left(\lim_{S\to \infty }\lim_{M\to \infty }D_{S}^{\left(M,n\right)}\left(A,\hat{f}\right)\right)\\ & = & \lim_{L\to \infty }\sum_{k=1}^{d}\lambda_{k}\left(\hat{f}\right)\\ & = & \sum_{k=1}^{d}\lambda_{k}\left(f\right). \end{array}$$

   □

According to Theorem 5, the EECD becomes the sum of all the LEs of a (piecewise) $C^{1}$ function *f* as *L*, *M*, and $S_{i,j}$ reach infinity.

At the end of this section, the relationship between the EECD and metric entropy is explained. Let *T* be a measurable transformation from *I* to *I*, preserving a probability measure μ on *I*, and ξ provides a measurable partition of *I*. Let $h_{\mu }\left(T,\xi \right)$ denote the metric entropy for the pair (μ,T) [17]. Subsequently, the EECD $D_{S}^{\left(M,n\right)}\left(\xi ,T\right)$ is equal to 0 for sufficiently large *M* and $S_{i,j}$ without depending on *n* [16]. Hence, the EECD $D_{S}^{\left(M\right)}\left(\xi ,T\right)$ is equal to or less than the metric entropy $h_{\mu }\left(T,\xi \right)$ for sufficiently large *M* and $S_{i,j}$.

## 4. Computational Algorithm of the EECD

In this section, by reviewing the derivation processes of the improved calculation formula of the EECD in [16], it is shown that all the LEs for an aperiodic orbit can be estimated by calculating the EECD.

To satisfy the relation in Theorem 5, the infinite values of *L*, *M*, and $S_{i,j}$ must be used. However, in the actual numerical computations of the EECD, these numbers must be set as finite values. Therefore, an improved calculation formula for the EECD was proposed in [16].

First, the derivation of the improved EECD calculation formula is reviewed for a stable periodic orbit. It is assumed that the map *f* creates a stable periodic orbit. Then, for any component $A_{i}\neq \emptyset$, there exists a component $A_{j_{i}}$ such that:

(15) $$|A_{j_{i}}\cap f\left(A_{i}\right)|=|f\left(A_{i}\right)\left|=\right|A_{j_{i}}|,$$

where |A| is the number of elements of the set *A*.

From Equation (15),

$$D_{S}^{\left(M,n\right)}\left(A,f\right)=\sum_{|A_{i}|>0}p_{i,A}^{\left(n\right)}\left(M\right)\log R\left(S_{i,j_{i}}\right),$$

because the conditional probability $p_{A}^{\left(n\right)}\left(j|i\right)\left(M\right)$ is expressed as

(16) $$p_{A}^{\left(n\right)}\left(j|i\right)\left(M\right)=\left\{\begin{array}{cc} 1 & \left(j=j_{i}\right)\\ 0 & \left(j\neq j_{i}\right) \end{array}\right..$$

Setting

$$\left(S_{i,j_{i}}\right)=\left\lfloor \sqrt[{d}]{\left|A_{i}\right|}\right\rfloor ,$$

then the following is obtained:

(17) $$R\left(S_{i,j_{i}}\right)≃\frac{\left|\left\{A_{i}:\;\left|A_{i}\right|>0\right\}\right|}{M}.$$

From Equation (17), it is evident that $R(S_{i,j_{i}})$ does not depend on $A_{i}$: Thus, the following improved calculation formula for the EECD for a stable orbit is obtained:

(18) $${\tilde{D}}_{S,1}^{\left(M,n\right)}\left(A,f\right)=\log\frac{\left|\left\{A_{i}:\;\left|A_{i}\right|>0\right\}\right|}{M}.$$

Second, the derivation process of the improved EECD calculation formula was reviewed for an aperiodic orbit. It is assumed that the map *f* does not create stable periodic orbits.

Let *L* and *M* be any sufficiently large natural numbers and let *m* be the Lebesgue measure on $R^{d}$. Let *f* be a piecewise linear function $\hat{f}$ given as Equation (10) under Assmption 1. Let us assume that *f* has the unique invariant measure μ.

Then, the following is obtained:

(19) $$\begin{array}{ccc} D_{S}^{\left(M,n\right)}\left(A,f\right) & = & \sum_{i=0}^{L^{d}-1}p_{i,A}^{\left(n\right)}\left(M\right)\left(\sum_{j=0}^{L^{d}-1}p_{A}^{\left(n\right)}\left(j|i\right)\left(M\right)\log\frac{R(S_{i,j})}{p_{A}^{\left(n\right)}\left(j|i\right)\left(M\right)}\right)\\ & ≃ & \sum_{i=0}^{L^{d}-1}\mu \left(f\left(A_{i}\right)\right)\left(\sum_{j=0}^{L^{d}-1}\frac{\mu (A_{j}\cap f\left(A_{i}\right))}{\mu (f\left(A_{i}\right))}\log\frac{\frac{m(A_{j}\cap f\left(A_{i}\right))}{m(A_{j})}}{\frac{\mu (A_{j}\cap f\left(A_{i}\right))}{\mu (f\left(A_{i}\right))}}\right)\\ & ≃ & \sum_{i=0}^{L^{d}-1}\sum_{j=0}^{L^{d}-1}\mu \left(A_{j}\cap f\left(A_{i}\right)\right)\log\frac{m(f\left(A_{i}\right))}{m(A_{j})}\\ & = & \sum_{i=0}^{L^{d}-1}p_{i,A}^{\left(n\right)}\left(M\right)\log\frac{m(f\left(A_{i}\right))}{m(A_{i})}. \end{array}$$

Here, the following relationship is used in the second approximation (Equation (19)).

$$\frac{\mu \left(A_{j}\cap f\left(A_{i}\right)\right)}{\mu \left(f(A_{i})\right)}≃\frac{m\left(A_{j}\cap f\left(A_{i}\right)\right)}{m\left(f(A_{i})\right)}.$$

For any set $X\;\left(\neq \emptyset \right)\subset I=\prod_{k=1}^{d}\left[a_{k},b_{k}\right]$,

$$\begin{array}{ccc} X & = & \left\{\left(x_{1},x_{2},\ldots ,x_{d}\right):\;x_{k}\in \left[a_{k},b_{k}\right],k=1,2,\ldots ,d\right\}\\ & = & \left\{\left({\left(x_{1}\right)}_{j},{\left(x_{2}\right)}_{j},\ldots ,{\left(x_{d}\right)}_{j}\right):\;{\left(x_{k}\right)}_{j}\in \left[a_{k},b_{k}\right],k=1,2,\ldots ,d,j=0,1,\ldots ,\left|X\right|-1\right\}. \end{array}$$

The variance–covariance matrix $\sum_{X}$ for all points x on *X*, is expressed as

$$\sum_{X}=\left(\begin{array}{cccc} {\left(\sigma_{1}^{2}\right)}_{X} & {\left(\sigma_{1,2}\right)}_{X} & \ldots & {\left(\sigma_{1,d}\right)}_{X}\\ {\left(\sigma_{2,1}\right)}_{X} & {\left(\sigma_{2}^{2}\right)}_{X} & \ldots & {\left(\sigma_{2,d}\right)}_{X}\\ \vdots & \vdots & \ddots & \vdots \\ {\left(\sigma_{d,1}\right)}_{X} & {\left(\sigma_{d,2}\right)}_{X} & \ldots & {\left(\sigma_{d}^{2}\right)}_{X} \end{array}\right),$$

where

$$\begin{array}{ccc} {\left(\sigma_{l,m}\right)}_{X} & = & \frac{1}{\left|X\right|}\sum_{j=0}^{|X|-1}\left({\left(x_{l}\right)}_{j}-{\bar{x}}_{l}\right)\left({\left(x_{m}\right)}_{j}-{\bar{x}}_{m}\right),\\ {\left(\sigma_{l}^{2}\right)}_{X} & = & \frac{1}{\left|X\right|}\sum_{j=0}^{|X|-1}{\left({\left(x_{l}\right)}_{j}-{\bar{x}}_{l}\right)}^{2},\\ {\left({\bar{x}}_{l}\right)}_{X} & = & \frac{1}{\left|X\right|}\sum_{j=0}^{|X|-1}{\left(x_{l}\right)}_{j}. \end{array}$$

Let ${\left(\lambda_{k}\right)}_{X}\;\left(k=1,2,\ldots ,d\right)$ be the eigenvalues of $\sum_{X}$ such that ${\left(\lambda_{i}\right)}_{X}\geq {\left(\lambda_{j}\right)}_{X}\;\left(i\geq j\right)$.

Thus, an improved calculation formula of the EECD for an aperiodic orbit is obtained as:

(20) $$\begin{array}{ccc} {\tilde{D}}_{S,2}^{\left(M,n\right)}\left(A,f\right) & = & \sum_{|A_{i}|>0}p_{i,A}^{\left(n\right)}\left(M\right)\log\frac{\prod_{k=1}^{d}\sqrt{{\left(\lambda_{k}\right)}_{f(A_{i})}}}{\prod_{k=1}^{d}\sqrt{{\left(\lambda_{k}\right)}_{A_{i}}}}. \end{array}$$

Thus, the improved calculation formula for the EECD is expressed as

(21) $${\tilde{D}}_{S}^{\left(M,n\right)}\left(A,f\right)\equiv \left\{\begin{array}{cc} {\tilde{D}}_{S,1}^{\left(M,n\right)}\left(A,f\right) & \left(\mathrm{when}\;\mathrm{the}\;\mathrm{map}\;f\;\mathrm{generates}\;a\;\mathrm{stable}\;\mathrm{periodic}\;\mathrm{orbit}\right)\\ {\tilde{D}}_{S,2}^{\left(M,n\right)}\left(A,f\right) & \left(\mathrm{otherwise}\right) \end{array}\right.$$

It is shown that all the LEs for an aperiodic orbit can be estimated for calculating the EECD as follows. Now, it is assumed that all the points x on $A_{i},f\left(A_{i}\right)$ are almost uniformly distributed over $C_{i},D_{i}$: see Equation (12)

Consider a random variable ξ that follows a uniform distribution on $\prod_{k=1}^{d}\left[c_{k},d_{k}\right]$. Subsequently, the standard deviation $\sigma_{k}$ of ξ is expressed as

(22) $$\sigma_{k}=\frac{1}{2\sqrt{3}}\left(d_{k}-c_{k}\right),\;k=1,2,\ldots ,d.$$

From Equation (22), the following is obtained:

(23) $$\begin{array}{ccc} & & d_{k}={\bar{x}}_{k}+\left(d_{k}˘{\bar{x}}_{k}\right)={\bar{x}}_{k}+\frac{1}{2}\left(d_{k}˘c_{k}\right)={\bar{x}}_{k}+\sqrt{3}\sigma_{k}, \end{array}$$

(24) $$\begin{array}{ccc} & & c_{k}={\bar{x}}_{k}˘\left({\bar{x}}_{k}˘c_{k}\right)={\bar{x}}_{k}-\frac{1}{2}\left(d_{k}-c_{k}\right)={\bar{x}}_{k}-\sqrt{3}\sigma_{k}. \end{array}$$

Let ${\left(u_{k}\right)}_{X}$ be the eigenvector corresponding to the eigenvalue ${\left(\lambda_{k}\right)}_{X}$, and

$${\langle x\rangle }_{X}\equiv {\left({\bar{x}}_{1},{\bar{x}}_{2},\ldots ,{\bar{x}}_{d}\right)}_{X}.$$

From Equations (23) and (24), $C_{i}$ and $D_{i}$ are expressed as:

(25) $$\begin{array}{ccc} C_{i} & = & \left\{{\langle x\rangle }_{A_{i}}+\sum_{k=1}^{d}\alpha_{k}\sqrt{{\left(\lambda_{k}\right)}_{A_{i}}}\frac{{\left(u_{k}\right)}_{A_{i}}}{∥{\left(u_{k}\right)}_{A_{i}}∥}:\;-\sqrt{3}\leq \alpha_{k}\leq \sqrt{3}\right\}, \end{array}$$

(26) $$\begin{array}{ccc} D_{i} & = & \left\{{\langle x\rangle }_{f(A_{i})}+\sum_{k=1}^{d}\beta_{k}\sqrt{{\left(\lambda_{k}\right)}_{f(A_{i})}}\frac{{\left(u_{k}\right)}_{f(A_{i})}}{∥{\left(u_{k}\right)}_{f(A_{i})}∥}:\;-\sqrt{3}\leq \beta_{k}\leq \sqrt{3}\right\}. \end{array}$$

Using Equations (25) and (26) yields:

(27) $$\begin{array}{ccc} & & m\left(A_{i}\right)=m\left(C_{i}\right)=\prod_{k=1}^{d}2\sqrt{3}\sqrt{{\left(\lambda_{k}\right)}_{A_{i}}}, \end{array}$$

(28) $$\begin{array}{ccc} & & m\left(f\left(A_{i}\right)\right)=m\left(D_{i}\right)=\prod_{k=1}^{d}2\sqrt{3}\sqrt{{\left(\lambda_{k}\right)}_{f(A_{i})}}. \end{array}$$

Furthermore, using Equations (27) and (28) the following is obtained:

(29) $$\frac{m(f\left(A_{i}\right))}{m(A_{i})}=\frac{\prod_{k=1}^{d}2\sqrt{3}\sqrt{{\left(\lambda_{k}\right)}_{f(A_{i})}}}{\prod_{k=1}^{d}2\sqrt{3}\sqrt{{\left(\lambda_{k}\right)}_{A_{i}}}}=\prod_{k=1}^{d}\frac{\sqrt{{\left(\lambda_{k}\right)}_{f(A_{i})}}}{\sqrt{{\left(\lambda_{k}\right)}_{A_{i}}}}.$$

From Equation (29),

(30) $$\begin{array}{ccc} \sum_{|A_{i}|>0}p_{i,A}^{\left(n\right)}\left(M\right)\log\frac{m(f\left(A_{i}\right))}{m(A_{i})} & = & \sum_{|A_{i}|>0}p_{i,A}^{\left(n\right)}\left(M\right)\log\prod_{k=1}^{d}\frac{\sqrt{{\left(\lambda_{k}\right)}_{f(A_{i})}}}{\sqrt{{\left(\lambda_{k}\right)}_{A_{i}}}}\\ & = & \sum_{|A_{i}|>0}p_{i,A}^{\left(n\right)}\left(M\right)\left(\sum_{k=1}^{d}\log\frac{\sqrt{{\left(\lambda_{k}\right)}_{f(A_{i})}}}{\sqrt{{\left(\lambda_{k}\right)}_{A_{i}}}}\right)\\ & = & \sum_{k=1}^{d}\left(\sum_{|A_{i}|>0}p_{i,A}^{\left(n\right)}\left(M\right)\log\frac{\sqrt{{\left(\lambda_{k}\right)}_{f(A_{i})}}}{\sqrt{{\left(\lambda_{k}\right)}_{A_{i}}}}\right). \end{array}$$

Now, let $r_{k}\left(x\right)$(k=1,2,…,d) be the eigenvalues of $\sqrt{Df^{t}\left(x\right)Df\left(x\right)}$ such that $r_{i}\left(x\right)\geq r_{j}\left(x\right)$(i≥j)(*k* = 1, 2, …, *d*) and let p(x) be the density function of x. Further, let $\left\{\lambda_{1}\left(f\right),\lambda_{2}\left(f\right),\ldots ,\lambda_{d}\left(f\right)\right\}$ be the Lyapunov spectrum of *f*. Then,

(31) $$\begin{array}{ccc} \sum_{|A_{i}|>0}p_{i,A}^{\left(n\right)}\left(M\right)\log\frac{m(f\left(A_{i}\right))}{m(A_{i})} & = & \sum_{|A_{i}|>0}\int_{A_{i}}\log\left(\prod_{k=1}^{d}r_{k}\left(x\right)\right)p\left(x\right)\prod_{l=1}^{d}dx_{l}\\ & = & \int_{a_{1}}^{b_{1}}\int_{a_{2}}^{b_{2}}\cdots \int_{a_{d}}^{b_{d}}\log\left(\prod_{k=1}^{d}r_{k}\left(x\right)\right)p\left(x\right)\prod_{l=1}^{d}dx_{l}\\ & = & \sum_{k=1}^{d}\int_{a_{1}}^{b_{1}}\int_{a_{2}}^{b_{2}}\cdots \int_{a_{d}}^{b_{d}}\log\left(r_{k}\left(x\right)\right)p\left(x\right)\prod_{l=1}^{d}dx_{l}\\ & = & \sum_{k=1}^{d}\lambda_{k}\left(f\right). \end{array}$$

Here the *k*th item (${EECD}_{k}$) of the EECD in Equation (30) is defined such that:

(32) $${\tilde{D}}_{S,2}^{\left(M,n\right)}\left(A,f,k\right)\equiv \sum_{|A_{i}|>0}p_{i,A}^{\left(n\right)}\left(M\right)\log\frac{\sqrt{{\left(\lambda_{k}\right)}_{f(A_{i})}}}{\sqrt{{\left(\lambda_{k}\right)}_{A_{i}}}},$$

where ${\tilde{D}}_{S,2}^{\left(M,n\right)}\left(A,f,k\right)\geq {\tilde{D}}_{S,2}^{\left(M,n\right)}\left(A,f,l\right)$(k≥l) and k,l∈{1,…,d}.

Further, using Equations (30) and (31), the following is obtained:

(33) $${\tilde{D}}_{S,2}^{\left(M,n\right)}\left(A,f,k\right)=\lambda_{k}\left(f\right).$$

Thus, the computational of the EECD for the map *f* is proposed as follows Algorithm 1:
**Algorithm 1:** Computational algorithm of the EECDStep 0. Consider a map *f* and create a partition $\left\{A_{i}\right\}$ in the following way:$$\begin{array}{ccc} & & I=\prod_{k=1}^{d}\left[a_{k},b_{k}\right]=\overset{L^{d}-1}{\underset{i=0}{⋃}}A_{i},\;A_{i}=\prod_{k=1}^{d}A_{i_{k}}^{\left(k\right)},\;i_{k}=0,1,\ldots ,L-1, \end{array}$$where
$$A_{i_{k}}^{\left(k\right)}=\left\{\begin{array}{cc} \left[a_{k}+\frac{b_{k}-a_{k}}{L}i_{k},\;a_{k}+\frac{b_{k}-a_{k}}{L}\left(i_{k}+1\right)\right) & (i_{k}=0,1,\ldots ,L-2),\\ \left[a_{k}+\frac{b_{k}-a_{k}}{L}\left(L-1\right),\;b_{k}\right] & \left(i_{k}=L-1\right) \end{array}\right.$$for any k=1,…,d.Step 1. Check whether the map *f* creates a stable periodic orbit.Step 2. If it does, then compute the EECD such that: $${\tilde{D}}_{S,1}^{\left(M,n\right)}\left(A,f\right)=\log\frac{\left|\left\{A_{i}:\;\left|A_{i}\right|>0\right\}\right|}{M}.$$Step 3. If not, then compute the ${\mathrm{EECD}}_{k}$ such that:$${\tilde{D}}_{S,2}^{\left(M,n\right)}\left(A,f,k\right)=\sum_{|A_{i}|>0}p_{i,A}^{\left(n\right)}\left(M\right)\log\frac{\sqrt{{\left(\lambda_{k}\right)}_{f(A_{i})}}}{\sqrt{{\left(\lambda_{k}\right)}_{A_{i}}}}$$for k=1,2,…,d, the process proceeds to Step 4.Step 4. Moreover, compute the EECD such that:$${\tilde{D}}_{S,2}^{\left(M,n\right)}\left(A,f\right)=\sum_{k=1}^{d}{\tilde{D}}_{S,2}^{\left(M,n\right)}\left(A,f,k\right).$$

## 5. Application of the Computational Algorithm of the EECD to Chaotic Dynamics

In this section, the computational algorithm of the EECD is applied to typical chaotic maps.

The essential basic elements for producing chaotic behavior are operations: “stretching” and “folding,” which are explained using a baker’s map as an example of a chaotic map.

The baker’s map *f* is defined as:

(34) $$f\left(x\right)=\left\{\begin{array}{cc} \left(2x_{1},\frac{1}{2}x_{2}\right)\; & \left(0\leq x_{1}\leq \frac{1}{2}\right)\\ \left(2x_{1}-1,\frac{1}{2}\left(x_{2}+1\right)\right)\; & \left(\frac{1}{2}<x_{1}\leq 1\right) \end{array}\right.,$$

where $x={\left(x_{1},x_{2}\right)}^{t}\in \left[0,1\right]\times \left[0,1\right]$,

The baker’s map *f* comprises two operations. In the first operation (stretching), the unit square was stretched twice in the $x_{1}$ direction and is compressed by half in the $x_{2}$ direction. Whereas, during the second operation (folding), the right part sticking out from the unit square was cut vertically and stacked on top of the left part.

Using the unit interval instead of the unit square, the Bernoulli shift map *f* is expressed as:

(35) $$f\left(x\right)=\left\{\begin{array}{cc} 2x\; & \left(0\leq x\leq \frac{1}{2}\right)\\ 2x-1\; & \left(\frac{1}{2}<x\leq 1\right) \end{array}\right.,$$

where x∈[0,1].

Thus, several typical one-dimensional chaotic maps exist with both the stretching and folding operations. In the next section, the computational algorithm of the EECD is applied for typical one and two-dimensional chaotic maps.

In general, the double type in the C language has been used for numerical computations. However, to ensure calculation accuracy, the floating-point type with a 1024-bit mantissa was used in the numerical computations of the eigenvalues of the variance-covariance matrix using GNU Multiprecision Library (GMP).

### 5.1. Application of the Computational Algorithm of the EECD to a One-Dimensional Chaotic Map

Consider a one-dimensional chaotic map f:I→I, where I=[a,b]. Let $\left\{A_{i}\right\}$ be the *L*-equipartition of *I* given as Equation (2) The improved formula of the EECD for a one-dimensional aperiodic map *f* is then expressed as:

$${\tilde{D}}_{S,2}^{\left(M,n\right)}\left(A,f\right)={\tilde{D}}_{S,2}^{\left(M,n\right)}\left(A,f,1\right),$$

where

(36) $${\tilde{D}}_{S,2}^{\left(M,n\right)}\left(A,f,1\right)=\sum_{|A_{i}|>0}p_{i,A}^{\left(n\right)}\left(M\right)\log\frac{\sqrt{{\left(\sigma_{1}^{2}\right)}_{f(A_{i})}}}{\sqrt{{\left(\sigma_{1}^{2}\right)}_{A_{i}}}}.$$

Here ${\left(\sigma_{1}^{2}\right)}_{X}$ is the variance of all points *x* on *X*.

In the following, M=100,000 and L=1000 are set.

#### 5.1.1. Numerical Computation Results for a Generalized Bernoulli Shift Map

In this section, the computational algorithm of the EECD is applied to a generalized Bernoulli shift map $f_{a}$ as the most straightforward one-dimensional chaotic map. The generalized Bernoulli shift map $f_{a}$ has derivative $\frac{df_{a}}{dx_{1}}\left(x_{1}\right)$ that depends only on parameter *a*.

The generalized Bernoulli shift map $f_{a}$ is defined as:

(37) $$f_{a}\left(x\right)=\left\{\begin{array}{cc} 2ax\; & \left(0\leq x\leq \frac{1}{2}\right)\\ a\left(2x-1\right)\; & \left(\frac{1}{2}<x\leq 1\right) \end{array}\right.,$$

where x∈[0,1] and 0≤a≤1. Then, the derivative $\frac{df_{a}}{dx}\left(x\right)$ of the generalized Bernoulli shift map $f_{a}$ is calculated as constant 2a. Thus, the LE of the generalized Bernoulli shift map $f_{a}$ was log2a.

Now, consider the orbit $\left\{x_{n}\right\}$ associated with the generalized Bernoulli shift map $f_{a}$ such that:

$$x_{n+1}=f_{a}\left(x_{n}\right),\;n=0,1,\ldots ,\;x_{0}=0.3333333.$$

**Figure 1:**
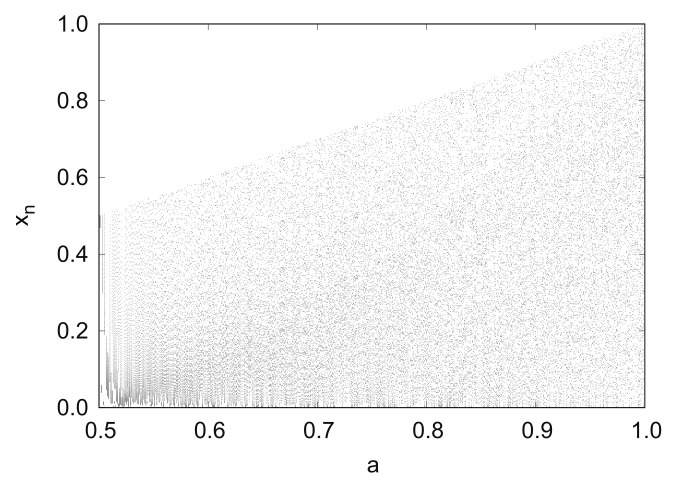
Bifurcation diagram of generalized Bernoulli shift map $f_{a}$.

Figure 1 shows the bifurcation diagram of the generalized Bernoulli shift map $f_{a}$ in 0.5≤a≤1.0. With an increase in parameter *a*, the points continue to spread over the entire unit interval.

**Figure 2:**
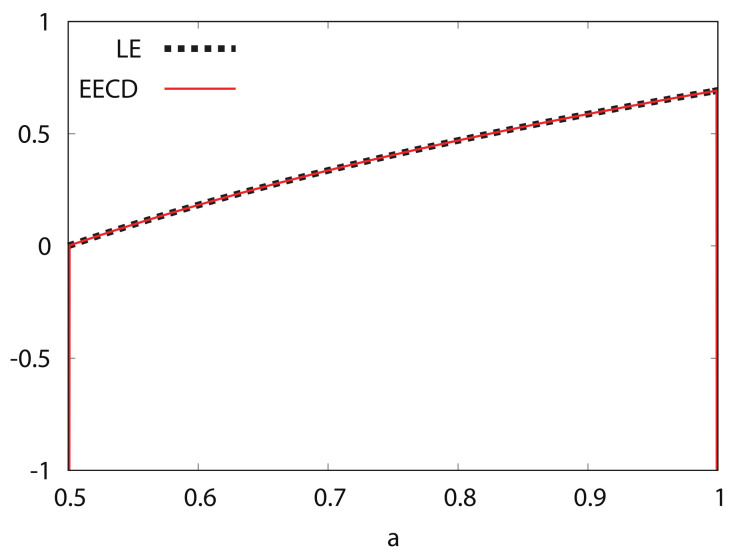
Lyapunov exponent (LE) and extended entropic chaos degree (EECD) versus *a* for generalized Bernoulli shift map $f_{a}$.

Figure 2 shows the numerical computation results for the LE $\lambda (f_{a})$ and the EECD ${\tilde{D}}_{S}^{\left(M,n\right)}\left(A,f_{a}\right)$ for the generalized Bernoulli shift map $f_{a}$. Comparisons of the EECD with the LE indicates that the EECD is approximately the same as the LE for the generalized Bernoulli shift map $f_{a}$.

#### 5.1.2. Numerical Computation Results for a Logistic Map

In this section, the computational algorithm of the EECD is applied to a logistic map $f_{a}$ as a typical one-dimensional chaotic map. The logistic map $f_{a}$ contains the derivative $\frac{df_{a}}{dx}\left(x\right)$ depending on *x* as well as parameter *a*.

The logistic map $f_{a}$ is defined as:

(38) $$f_{a}\left(x\right)=ax\left(1-x\right)$$

where x∈[0,1] and 3.5≤a≤4.0. Then, the derivative $\frac{df_{a}}{dx}\left(x\right)$ of the logistic map $f_{a}$ was calculated as a(1−2x). Thus, $\frac{df_{a}}{dx_{1}}\left(x\right)$ depends on both parameters *a* and *x*.

Now, consider the orbit $\left\{x_{n}\right\}$ associated with logistic map $f_{a}$ such that

$$x_{n+1}=f_{a}\left(x_{n}\right),\;n=0,1,\ldots ,\;x_{0}=0.3333333.$$

**Figure 3:**
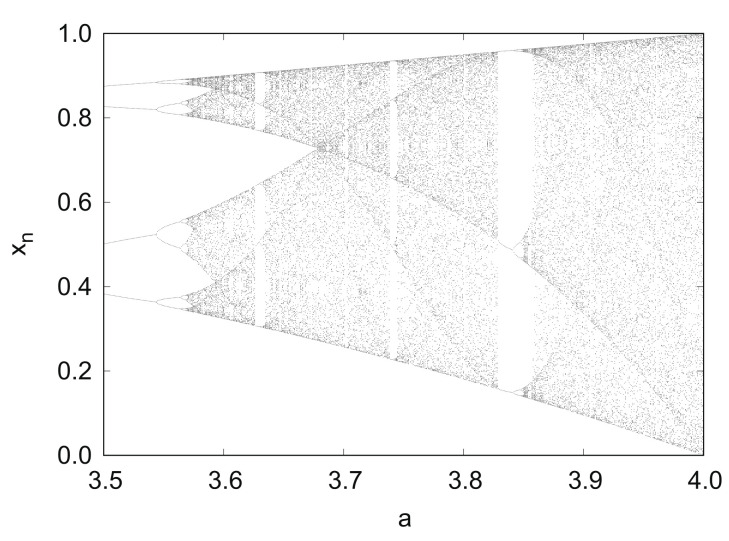
Bifurcation diagram of logistic map $f_{a}$.

Figure 3 shows the bifurcation diagram of the logistic map $f_{a}$ in 3.5≤a≤4.0.

**Figure 4:**
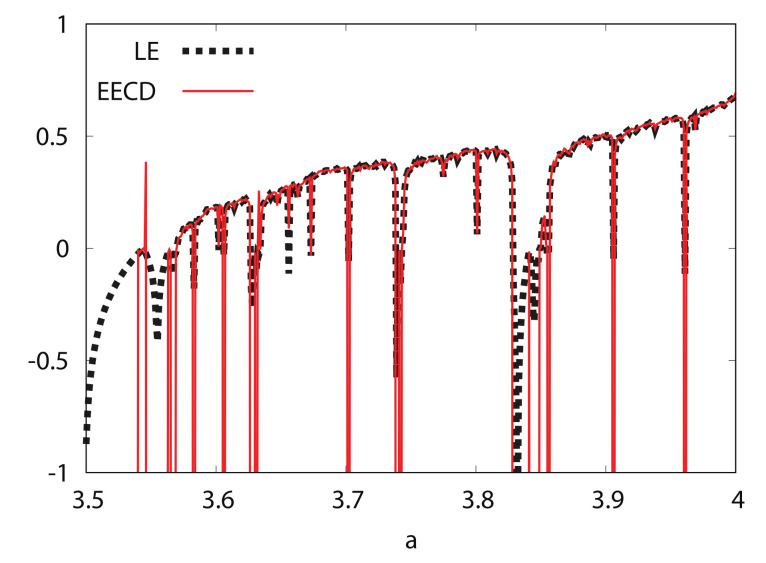
LE and EECD versus *a* for logistic map $f_{a}$.

Figure 4 shows the numerical computation results for the LE $\lambda (f_{a})$ and EECD ${\tilde{D}}_{S}^{\left(M,n\right)}\left(A,f_{a}\right)$ for the logistic map $f_{a}$. Comparing the EECD with the LE, the EECD is approximately the same as the LE for the logistic map $f_{a}$, except for several *a*s, where the orbit of $f_{a}$ is periodic.

### 5.2. Application of the Computational Algorithm of the EECD to a Two-Dimensional Chaotic Map

Consider a two-dimensional chaotic map f:I→I, where $I=\left[a_{1},b_{1}\right]\times \left[a_{2},b_{2}\right]$. Let $\left\{A_{i}\right\}$ be the $L^{2}$-equipartition of *I*given as Equation (7) at d=2. Then, the improved formula of the EECD for a two-dimensional aperiodic map *f* is expressed as:

$${\tilde{D}}_{S,2}^{\left(M,n\right)}\left(A,f\right)=\sum_{k=1}^{2}{\tilde{D}}_{S,2}^{\left(M,n\right)}\left(A,f,k\right),$$

where

(39) $${\tilde{D}}_{S,2}^{\left(M,n\right)}\left(A,f,k\right)=\sum_{|A_{i}|>0}p_{i,A}^{\left(n\right)}\left(M\right)\log\frac{\sqrt{{\left(\lambda_{k}\right)}_{f(A_{i})}}}{\sqrt{{\left(\lambda_{k}\right)}_{A_{i}}}}.$$

Here, ${\left(\lambda_{k}\right)}_{X}\;\left(k=1,2\right)$ are the eigenvalues of $\sum_{X}$ such that: ${\left(\lambda_{1}\right)}_{X}\geq {\left(\lambda_{2}\right)}_{X}$. The variance–covariance matrix $\sum_{X}$ for all points x on *X*, is expressed as:

(40) $$\sum_{X}=\left(\begin{array}{cc} {\left(\sigma_{1}^{2}\right)}_{X} & {\left(\sigma_{1,2}\right)}_{X}\\ {\left(\sigma_{2,1}\right)}_{X} & {\left(\sigma_{2}^{2}\right)}_{X} \end{array}\right).$$

The eigenvalues of $\sum_{X}$ can be expressed as those numbers λ such that: $\left|\lambda I-\sum_{X}\right|=0$. Using Equation (40), the following is obtained:

(41) $$\lambda =\frac{{\left(\sigma_{1}^{2}\right)}_{X}+{\left(\sigma_{2}^{2}\right)}_{X}\pm \sqrt{{\left(\sigma_{1}^{2}\right)}_{X}^{2}+{\left(\sigma_{2}^{2}\right)}_{X}^{2}-2{\left(\sigma_{1}^{2}\right)}_{X}^{2}{\left(\sigma_{2}^{2}\right)}_{X}^{2}+4{\left(\sigma_{1,2}\right)}_{X}^{2}}}{2}.$$

Because ${\left(\lambda_{1}\right)}_{X}\geq {\left(\lambda_{2}\right)}_{X}$, the following is true:

(42) $$\begin{array}{ccc} {\left(\lambda_{1}\right)}_{X} & = & \frac{{\left(\sigma_{1}^{2}\right)}_{X}+{\left(\sigma_{2}^{2}\right)}_{X}+\sqrt{{\left(\sigma_{1}^{2}\right)}_{X}^{2}+{\left(\sigma_{2}^{2}\right)}_{X}^{2}-2{\left(\sigma_{1}^{2}\right)}_{X}^{2}{\left(\sigma_{2}^{2}\right)}_{X}^{2}+4{\left(\sigma_{1,2}\right)}_{X}^{2}}}{2}, \end{array}$$

(43) $$\begin{array}{ccc} {\left(\lambda_{2}\right)}_{X} & = & \frac{{\left(\sigma_{1}^{2}\right)}_{X}+{\left(\sigma_{2}^{2}\right)}_{X}-\sqrt{{\left(\sigma_{1}^{2}\right)}_{X}^{2}+{\left(\sigma_{2}^{2}\right)}_{X}^{2}-2{\left(\sigma_{1}^{2}\right)}_{X}^{2}{\left(\sigma_{2}^{2}\right)}_{X}^{2}+4{\left(\sigma_{1,2}\right)}_{X}^{2}}}{2}. \end{array}$$

In the following, M=1,000,000 and $L^{2}=1000^{2}$ are set.

#### 5.2.1. Numerical Computation Results for a Generalized Baker’s Map

In this section, the computational algorithm of the EECD is applied to a generalized baker’s map $f_{a}$ as one of the simplest two-dimensional chaotic maps. The generalized baker’s map $f_{a}$ has Jacobian matrices $Df_{a}\left(x\right)$ that depend only on parameter *a*. In addition, its determinant $detDf_{a}\left(x\right)$ is also only dependent on parameter *a*.

The generalized baker’s map $f_{a}$ is defined as follows:

(44) $$f_{a}\left(x\right)=\left\{\begin{array}{cc} \left(2ax_{1},\frac{1}{2}ax_{2}\right)\; & \left(0\leq x_{1}\leq \frac{1}{2}\right)\\ \left(a\left(2x_{1}-1\right),\frac{1}{2}a\left(x_{2}+1\right)\right)\; & \left(\frac{1}{2}<x_{1}\leq 1\right) \end{array}\right.,$$

where $x={\left(x_{1},x_{2}\right)}^{t}\in \left[0,1\right]\times \left[0,1\right]$ and 0≤a≤1. Then, the Jacobian matrix of the baker’s map $f_{a}$ is calculated as:

(45) $$Df_{a}\left(x\right)=\left(\begin{array}{cc} 2a & 0\\ 0 & \frac{1}{2}a \end{array}\right).$$

Thus, $Df_{a}\left(x\right)$ depends only on parameter *a*. The dynamics associated with the generalized baker’s map $f_{a}$ are dissipative for 0≤a<1, because $|detDf_{a}\left(x\right)|=a^{2}$ [18].

Now, consider the orbit $\left\{x_{n}\right\}$ associated with the generalized baker map $f_{a}$ such that:

$$x_{n+1}=f_{a}\left(x_{n}\right),\;n=0,1,2,\ldots ,x_{0}={\left(0.3333,0.3333\right)}^{t}.$$

Let $f_{a}^{m}$ be the transformation from $dv_{0}$ to $dv_{m}$ on $R^{2}$. This directly yields:

(46) $$dv_{m}=detDf_{a}^{m}\left(v_{0}\right)dv_{0}=a^{2m}dv_{0}.$$

Thus,

(47) $$\lambda_{1}\left(f_{a}\right)+\lambda_{2}\left(f_{a}\right)=\lim_{m\to \infty }\frac{1}{m}\log\left|\frac{dv_{m}}{dv_{0}}\right|=\lim_{m\to \infty }\frac{\log a^{2m}}{m}=2\log a$$

**Figure 5:**
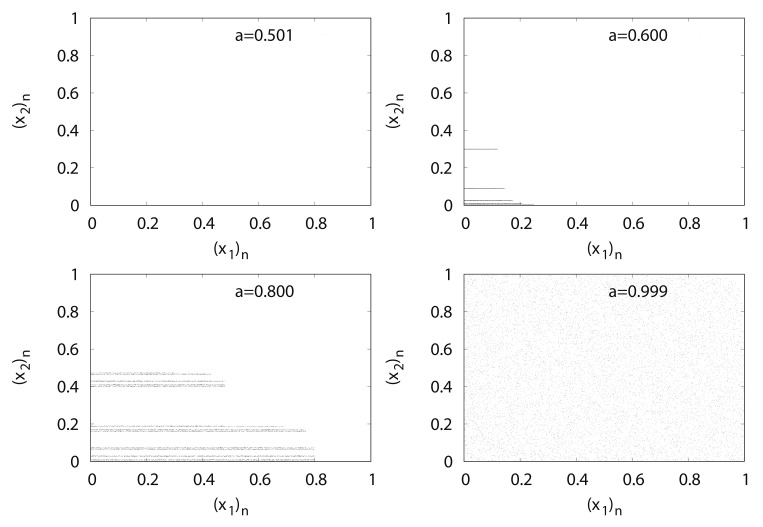
${\left(x_{2}\right)}_{n}$ versus ${\left(x_{1}\right)}_{n}$ for generalized baker’s map $f_{a}$.

Figure 5 shows the typical orbits of the generalized baker’s map $f_{a}$. With an increase in parameter *a*, points spread from certain lines over to the entire unit square.

**Figure 6:**
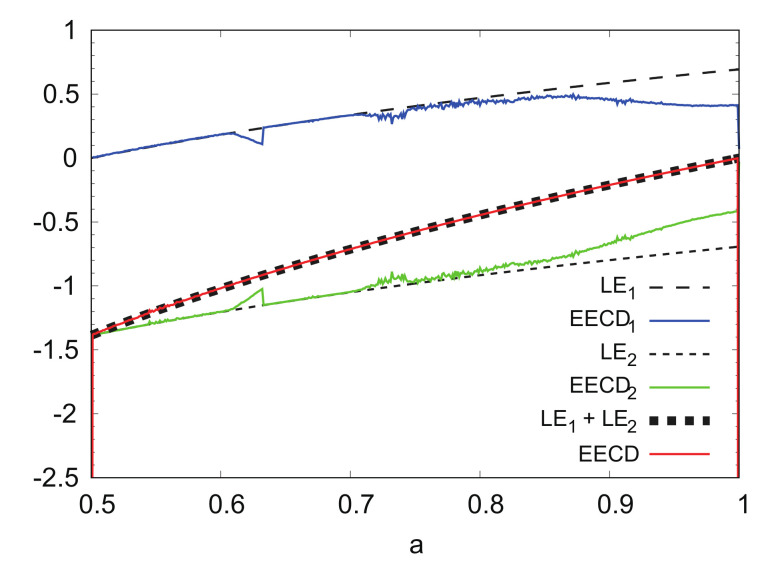
${LE}_{k}$, ${EECD}_{k}$(k=1,2), ${LE}_{1}+{LE}_{2}$, and EECD versus *a* for generalized baker’s map $f_{a}$.

Figure 6 shows the numerical computation results for the *k*-th LE (${LE}_{k}$) $\lambda_{k}\left(f_{a}\right)$, ${EECD}_{k}$${\tilde{D}}_{S,2}^{\left(M,n\right)}\left(A,f_{a},k\right)$(k=1,2) of the EECD, and total sum (${LE}_{1}+{LE}_{2}$) $\lambda_{1}\left(f_{a}\right)+\lambda_{2}\left(f_{a}\right)$ of the LEs and EECD for the generalized baker’s map $f_{a}$. Comparisons of the EECD with ${LE}_{1}+{LE}_{2}$ indicate that the EECD is approximately the same as ${LE}_{1}+{LE}_{2}$ for the generalized baker map $f_{a}$. The same is true for the ${EECD}_{k}$ and ${LE}_{k}$ for k=1,2 in 0.5≤a≤0.8. However, as parameter *a* increases in 0.8<a≤1.0, the difference between ${EECD}_{k}$ and ${LE}_{k}$ for k=1,2, increases.

With an increase in parameter *a* in 0.8≤a≤1.0, the shape of the domain of the points included in $A_{i}$ changes from multiple lines over the entire plane. Considering this feature, increasing the number *M* of points was considered because the number *M* of points may not be sufficient to cover the entire region at M=1,000,000.

**Figure 7:**
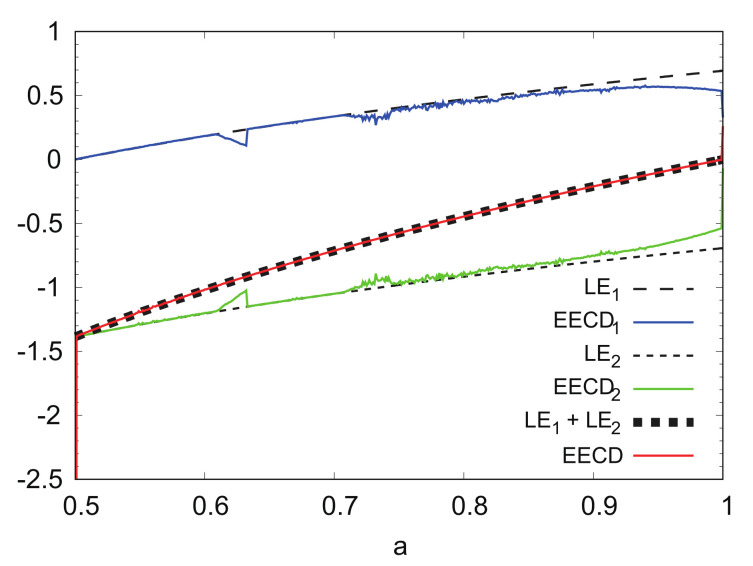
${\mathrm{LE}}_{k}$, ${\mathrm{EECD}}_{k}$(k=1,2), ${\mathrm{LE}}_{1}+{\mathrm{LE}}_{2}$, and EECD versus *a* for generalized baker’s map $f_{a}$ (M=10,000,000).

Figure 7 shows the numerical computation results for ${LE}_{k}$, ${EECD}_{k}$(k=1,2), ${LE}_{1}+{LE}_{2}$, and EECD at M=10,000,000 instead of M=1,000,000. By increasing the number of points *M*, the difference between ${EECD}_{k}$ and ${LE}_{k}$ for k=1,2, was reduced to 0.8≤a≤1.0.

For any two-dimensional chaotic map *f*, the average expansion rate in the stretching of *f* and the average contraction rate during the folding of *f* correspond to $\exp(\lambda_{1}\left(f\right))$ and $\exp(\lambda_{2}\left(f\right))$, respectively.

#### 5.2.2. Numerical Computation Results for a Tinkerbell Map

In this section, the computational algorithm of the EECD is applied to a Tinkerbell map $f_{a}$ as a two-dimensional dissipative chaotic map [18]. The Jacobian matrix $Df_{a}\left(x\right)$ of the Tinkerbell mapping $f_{a}$ depends on x and parameter *a*. The same is true for its determinant $detDf_{a}\left(x\right)$.

The Tinkerbell map $f_{a}$ is defined as:

(48) $$f_{a}\left(x\right)={\left(x_{1}^{2}-x_{2}^{2}+ax_{1}-0.6013x_{2},2x_{1}x_{2}+2x_{1}+0.5x_{2}\right)}^{t},$$

where $x={\left(x_{1},x_{2}\right)}^{t}\in \left[-1.3,0.5\right]\times \left[-1.6,0.6\right]$ for 0.7≤a≤0.9.

The Jacobian matrix of the Tinkerbell map $f_{a}$ is calculated as:

(49) $$Df_{a}\left(x\right)=\left(\begin{array}{cc} 2x_{1}+a & -2x_{2}-0.6013\\ 2x_{2}+2 & 2x_{1}+0.5 \end{array}\right).$$

Thus, $Df_{a}\left(x\right)$ depends on x and parameter *a*.

Now, consider the orbit $\left\{x_{n}\right\}$ associated with the Tinkerbell map $f_{a}$, such that:

$$x_{n+1}=f_{a}\left(x_{n}\right),\;n=0,1,2,\ldots ,x_{0}={\left(0.1,0.1\right)}^{t}.$$

**Figure 8:**
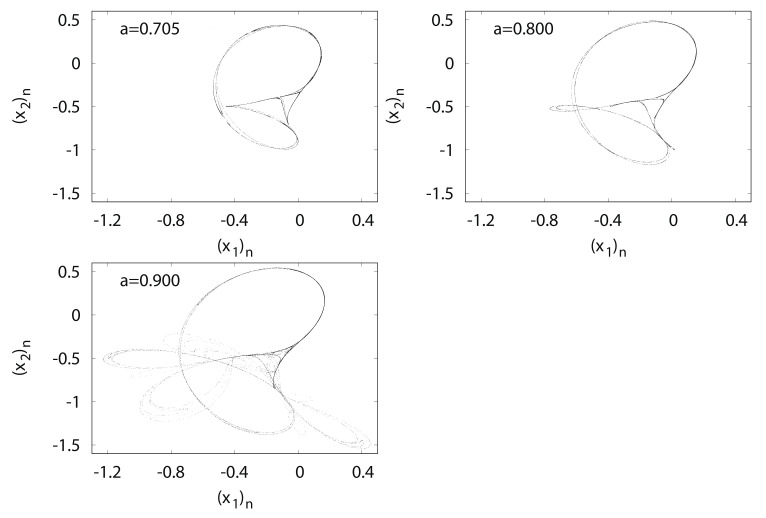
${\left(x_{2}\right)}_{n}$ versus ${\left(x_{1}\right)}_{n}$ for Tinkerbell map $f_{a}$.

Figure 8 shows typical orbits of the Tinkerbell map $f_{a}$. The trajectory of the Tinkerbell map $f_{a}$ draws an unusual attractor at a=0.9. The origin of the name of the Tinkerbell map $f_{a}$ is based on the shape of a strange attractor that appears similar to the movement of a fairy named Tinker Bell, who appeared in a Disney film.

**Figure 9:**
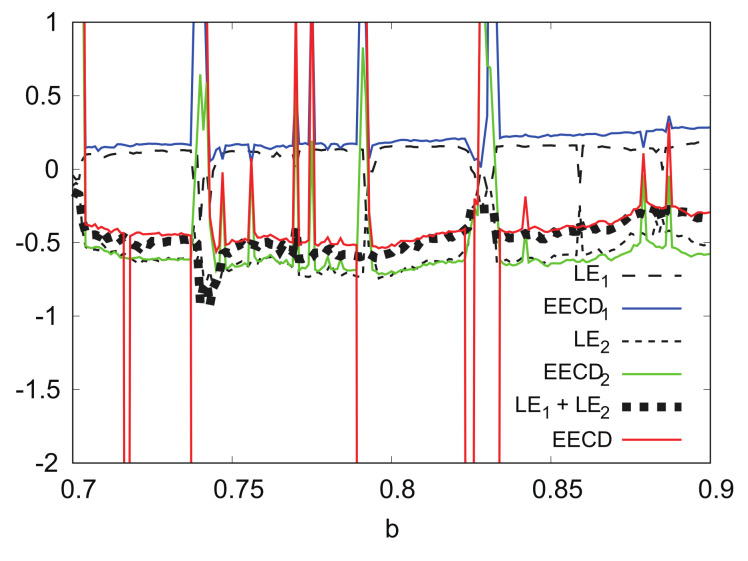
${LE}_{k}$, ${EECD}_{k}$(k=1,2), ${LE}_{1}+{LE}_{2}$, and EECD versus *a* for Tinkerbell map $f_{a}$.

Figure 9 shows the numerical computation results for ${LE}_{k}$, ${EECD}_{k}$(k=1,2), ${LE}_{1}+{LE}_{2}$, and the EECD for the Tinkerbell map $f_{a}$. Comparisons of the EECD with ${LE}_{1}+{LE}_{2}$ indicate that EECD is approximately the same as ${LE}_{1}+{LE}_{2}$ for the Tinkerbell map $f_{a}$ in 0.7≤a≤0.9, except for several *a*s, where the orbit of $f_{a}$ is periodic. The same is true for the ${EECD}_{k}$ and ${LE}_{k}$ for k=1,2.

#### 5.2.3. Numerical Computation Results for an Ikeda Map

In this section, the computational algorithm of the EECD is applied to an Ikeda map $f_{a}$ as a two-dimensional dissipative chaotic map [18]. The Ikeda map $f_{a}$ contains the Jacobian matrix $Df_{a}\left(x\right)$ that depends on x and parameter *a*. However, its determinant $detDf_{a}\left(x\right)$ depends only on parameter *a*.

The modified Ikeda map is expressed as a complex map in [19,20]:

(50) $$f\left(z\right)=A+Bze^{iK/(|z{|}^{2}+1)+C},\;z\in C,\;\;A,B,K,C\in R.$$

The Ikeda map $f_{a}$ is defined as a real two-dimensional example of Equation (50) as:

(51) $$f_{a}\left(x\right)={\left(1+a\left(x_{1}\cos t-x_{2}\sin t\right),a\left(x_{1}\sin t+x_{2}\cos t\right)\right)}^{t},$$

where

$$t=0.4-\frac{6}{1+x_{1}^{2}+x_{2}^{2}}$$

and $x={\left(x_{1},x_{2}\right)}^{t}\in \left[-0.4,1.8\right]\times \left[-2.3,0.9\right]$ for 0.7≤a≤0.9.

The Jacobian matrix of the Ikeda map $f_{a}$ is calculated as:

(52) $$Df_{a}\left(x\right)=a\left(\begin{array}{cc} u_{1}\cos t-u_{2}\sin t & -u_{3}\sin t-u_{4}\cos t\\ u_{1}\sin t+u_{2}\cos t & u_{3}\cos t-u_{4}\sin t \end{array}\right),$$

where

$$\begin{array}{ccc} & & u_{1}=1-\frac{12x_{1}x_{2}}{{\left(1+x_{1}^{2}+x_{2}^{2}\right)}^{2}},\;\;u_{2}=\frac{12x_{1}^{2}}{{\left(1+x_{1}^{2}+x_{2}^{2}\right)}^{2}}\\ & & u_{3}=1+\frac{12x_{1}x_{2}}{{\left(1+x_{1}^{2}+x_{2}^{2}\right)}^{2}},\;\;u_{4}=\frac{12x_{2}^{2}}{{\left(1+x_{1}^{2}+x_{2}^{2}\right)}^{2}}. \end{array}$$

Thus, $Df_{a}\left(x\right)$ depends on x and parameter *a*. Further, the dynamics associated with the Ikeda map $f_{a}$ are dissipative for 0≤a<1, because $|detDf_{a}\left(x\right)|=a^{2}$.

Now, consider the orbit $\left\{x_{n}\right\}$ associated with the Ikeda map $f_{a}$ such that:

$$x_{n+1}=f_{a}\left(x_{n}\right),\;n=0,1,2,\ldots ,x_{0}={\left(0.1,0.0\right)}^{t}.$$

Let $f_{a}^{m}$ be the transformation from $dv_{0}$ to $dv_{m}$ on $R^{2}$. By using the chain rule and $\det Df_{a}\left(x\right)=a^{2}$ for the Ikeda map $f_{a}$, the following equation is obtained:

(53) $$dv_{m}=detDf_{a}^{m}\left(v_{0}\right)dv_{0}=a^{2m}dv_{0}.$$

Thus,

(54) $$\lambda_{1}\left(f_{a}\right)+\lambda_{2}\left(f_{a}\right)=\lim_{m\to \infty }\frac{1}{m}\log\left|\frac{dv_{m}}{dv_{0}}\right|=\lim_{m\to \infty }\frac{\log a^{2m}}{m}=2\log a.$$

**Figure 10:**
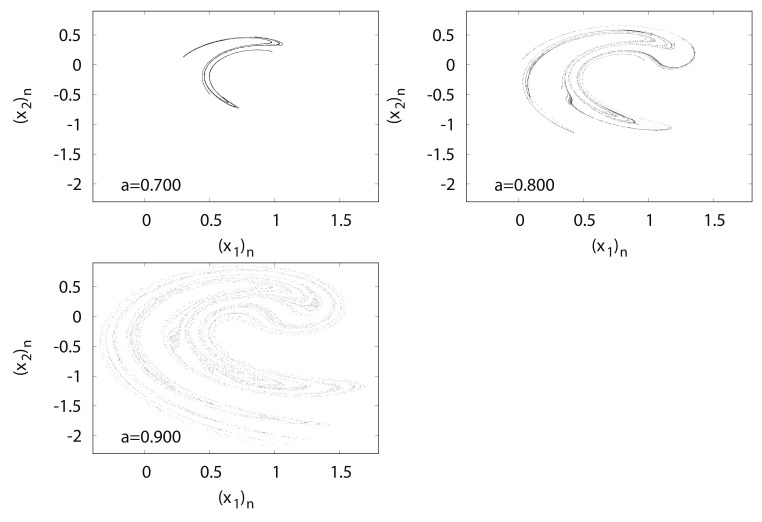
${\left(x_{2}\right)}_{n}$ versus ${\left(x_{1}\right)}_{n}$ for Ikeda map $f_{a}$.

Figure 10 shows typical orbits of the Ikeda map $f_{a}$. With an increase in parameter *a*, the attractor generated by the Ikeda map $f_{a}$ grows in size. Moreover, regarding the $f_{a}$ plots, the Ikeda map might be conjugate to a Hénon map [21].

**Figure 11:**
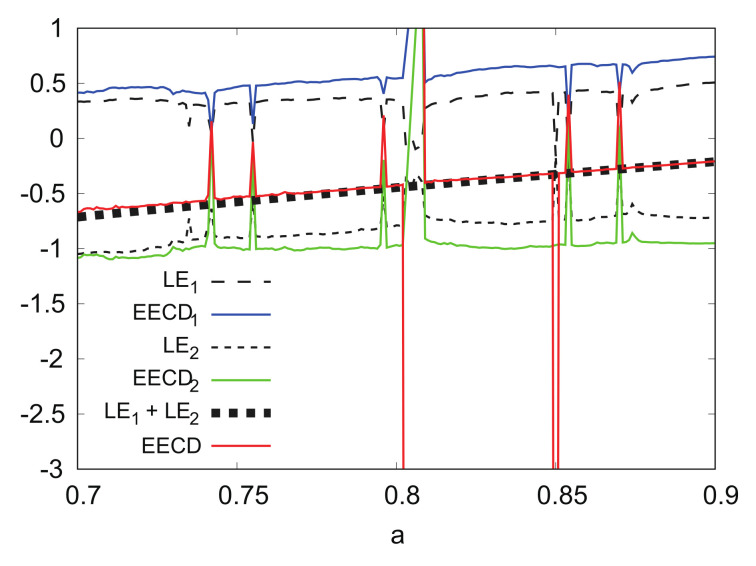
${LE}_{k}$, ${EECD}_{k}$(k=1,2), ${LE}_{1}+{LE}_{2}$, and EECD versus *a* for Ikeda map $f_{a}$.

Figure 11 shows the numerical computation results for ${LE}_{k}$, ${EECD}_{k}$(k=1,2), ${LE}_{1}+{LE}_{2}$, and the EECD for the Ikeda map $f_{a}$. Comparisons of the EECD with the ${LE}_{1}+{LE}_{2}$ indicate that the EECD is approximately the same as ${LE}_{1}+{LE}_{2}$ for the Ikeda map $f_{a}$, except for several values of *a*, where the orbit of $f_{a}$ is periodic. However, there is a small difference between ${EECD}_{k}$ and ${LE}_{k}$ for k=1,2, in 0.7≤a≤0.9. These differences cannot necessarily decrease, even if the number *M* of points and the number $L^{2}$ of all the components of the equipartition of *I* are increased.

This problem may be related to the shape of the trajectory generated by the Ikeda map $f_{a}$. The shape of the minimum region, including all the points in $A_{i}$ of *I* for the Ikeda map $f_{a}$, is a partial spiral. However, the region above is regarded as a rectangle $C_{i}$ (Equation (25)), as evident in the computational algorithm of the EECD. This region above the EECD may cause the difference between ${EECD}_{k}$ and ${LE}_{k}$ for k=1,2.

#### 5.2.4. Numerical Computation Results for a Hénon Map

In this section, the computational algorithm of the EECD is applied to a Hénon map $f_{a,b}$ as a two-dimensional dissipative chaotic map. The Hénon map $f_{a,b}$ has the Jacobian matrix $Df_{a,b}\left(x\right)$, which is dependent on x and parameter *b*. However, its determinant $detDf_{a,b}\left(x\right)$ depends only on parameter *b*.

The Hénon map $f_{a,b}$ is defined as:

(55) $$f_{a,b}\left(x\right)={\left(a-x_{1}^{2}+bx_{2},x_{1}\right)}^{t},$$

where $x={\left(x_{1},x_{2}\right)}^{t}\in {\left[-1.8,1.8\right]}^{2}$ for a=1.4,0<b≤0.3.

In the following section, $f_{1.4,b}=f_{b}$ is rewritten.

The Jacobian matrix of the Hénon map $f_{a,b}$ is calculated as follows:

(56) $$Df_{a,b}\left(x\right)=\left(\begin{array}{cc} 2x_{1} & b\\ 1 & 0 \end{array}\right).$$

Thus, $Df_{a,b}\left(x\right)$ depends on $x_{1}$ and parameter *b*. Further, dynamics associated with the Hénon map $f_{a,b}$ are dissipative at 0≤b<1, because $|detDf_{a,b}\left(x\right)|=b$.

Now, consider the orbit $\left\{x_{n}\right\}$ associated with the Hénon map $f_{b}$ such that:

$$x_{n+1}=f_{b}\left(x_{n}\right),\;n=0,1,2,\ldots ,\;x_{0}={\left(0.1,0.1\right)}^{t}.$$

Let $f_{b}^{m}$ be the transformation from $dv_{0}$ to $dv_{m}$ on $R^{2}$. By using the chain rule and $detDf_{b}\left(x\right)=-b$ for the Hénon map $f_{b}$, the following is obtained:

(57) $$dv_{m}=detDf_{b}^{m}\left(v_{0}\right)dv_{0}={\left(-b\right)}^{m}dv_{0}.$$

Thus,

(58) $$\lambda_{1}\left(f_{b}\right)+\lambda_{2}\left(f_{b}\right)=\lim_{m\to \infty }\frac{1}{m}\log\left|\frac{dv_{m}}{dv_{0}}\right|=\lim_{m\to \infty }\frac{\log b^{m}}{m}=\log b.$$

**Figure 12:**
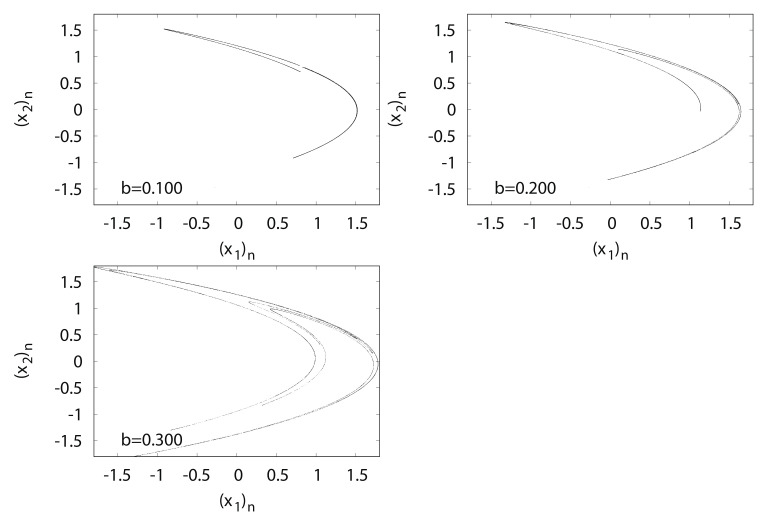
${\left(x_{2}\right)}_{n}$ versus ${\left(x_{1}\right)}_{n}$ for Hėnon map $f_{b}$.

Figure 12 shows typical orbits of the Hénon map $f_{b}$. The trajectory of the Hénon attractor exhibits a fractal structure such that upon expanding the strip region, innumerable parallel curves reappear in the strip.

**Figure 13:**
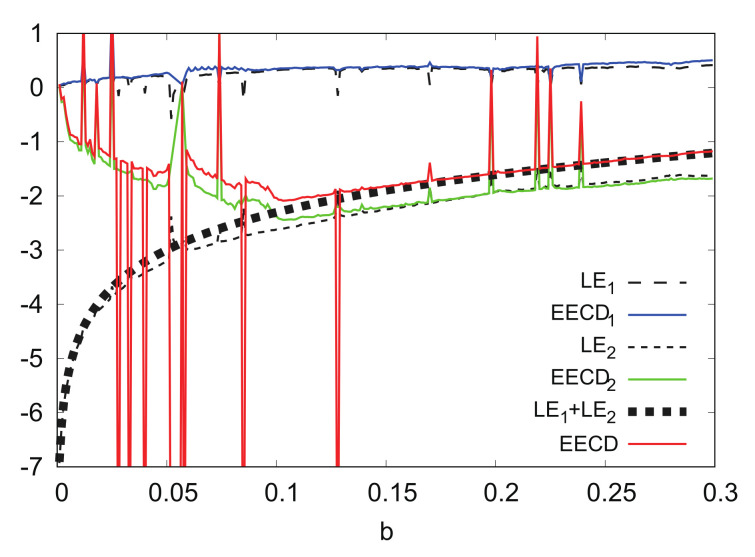
${LE}_{k}$, ${EECD}_{k}$(k=1,2), ${LE}_{1}+{LE}_{2}$, and EECD versus *b* for Hėnon map $f_{b}$.

Figure 13 shows the numerical computation results for ${LE}_{k}$, ${EECD}_{k}$(k=1,2), ${LE}_{1}+{LE}_{2}$, and EECD for the Hénon map $f_{b}$. Comparisons of the ${EECD}_{1}$ and ${\mathrm{LE}}_{1}$ indicate that ${EECD}_{1}$ is approximately the same as ${LE}_{1}$ for the Hénon map $f_{b}$, except for several *b*s, where the orbit of the Hénon map $f_{b}$ is periodic. The same is true for the ${EECD}_{2}$ and ${LE}_{2}$, as well as for the EECD and ${LE}_{1}+{LE}_{2}$ in 0.1≤b≤0.3. However, there was a remarkable difference between ${EECD}_{2}$ and ${LE}_{2}$ in 0≤b<0.1, the EECD attained noticeably different values from ${LE}_{1}+{LE}_{2}$ for 0≤b<0.1. The orbit of the map $f_{b}$ is not periodic at 0.0≤b<0.1.

Now, consider another expression for the smaller eigenvalue ${\left(\lambda_{2}\right)}_{X}$ among the eigenvalues of the variance-covariance matrix $\sum_{X}$ such that:

(59) $$\begin{array}{ccc} {\left(\lambda_{2}\right)}_{X} & = & \frac{{\left(\sigma_{1}^{2}\right)}_{X}+{\left(\sigma_{2}^{2}\right)}_{X}-\sqrt{{\left\{{\left(\sigma_{1}^{2}\right)}_{X}+{\left(\sigma_{2}^{2}\right)}_{X}\right\}}^{2}-4{\left(\sigma_{1}^{2}\right)}_{X}{\left(\sigma_{2}^{2}\right)}_{X}\left\{1-{\left(\rho_{X}\right)}^{2}\right\}}}{2}, \end{array}$$

where $\rho_{X}$ is the autocorrelation function for all points x on component *X*.

From Equation (59), if the absolute value of $\rho_{X}$ is equal to 1, then

$${\left(\lambda_{2}\right)}_{X}=0.$$

The ratio of ${\left(\lambda_{2}\right)}_{f(A_{i})}$ to ${\left(\lambda_{2}\right)}_{A_{i}}$ is included in the formula: ${EECD}_{2}$ (Equation (39)). Thus, if the absolute value of $\rho_{A_{i}}$ is almost equal to 1, then accurately computing ${EECD}_{2}$ is challenging because ${\left(\lambda_{2}\right)}_{f(A_{i})}$ must be divided by ${\left(\lambda_{2}\right)}_{A_{i}}$, close to 0.

Let E(|ρ|) be the average of $|\rho_{A_{i}|}$, such that:

(60) $$E\left(\left|\rho \right|\right)=\sum_{|A_{i}|>3}\frac{|A_{i}|}{\sum_{|A_{i}|>3}\left|A_{i}\right|}\left|\rho_{A_{i}}\right|.$$

**Figure 14:**
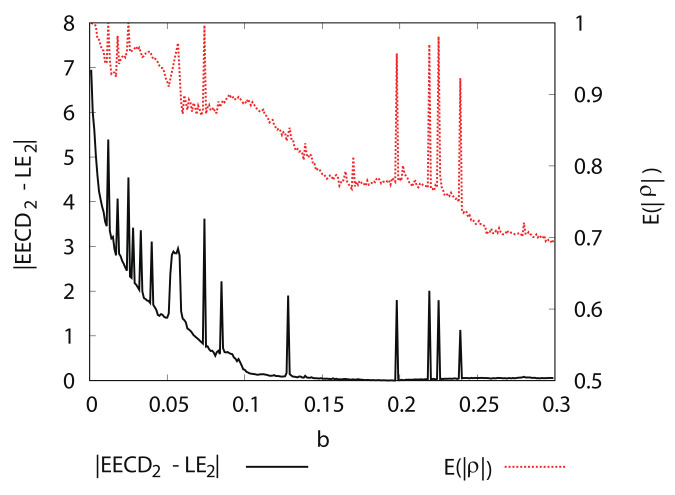
$|{EECD}_{2}-{LE}_{2}|$ and $E\left(\left|\rho \right|\right)$ versus *b* for Hėnon map $f_{b}$.

Figure 14 shows the numerical computation results for $|{EECD}_{2}-{LE}_{2}|$, and E(|ρ|) for the Hénon map $f_{b}$. E(|ρ|) is very close to 1 in 0≤b<0.1. Therefore, it can be concluded that the remarkable difference between the ${EECD}_{2}$ and ${LE}_{2}$ is caused by $|\rho_{A_{i}}|≐1$, for any $A_{i}$.

#### 5.2.5. Numerical Computation Results for a Standard Map

In this section, the computational algorithm of the EECD is applied to a standard map $f_{K}$ as a two-dimensional conservative chaotic map. The standard map $f_{K}$ has the Jacobian matrix $Df_{K}\left(y\right)$, which is dependent on y and parameter *K*. However, its determinant $detDf_{K}\left(x\right)$ remains constant at 1.

The standard map $f_{K}$ is defined as:

(61) $$f_{K}\left(y\right)={\left(\theta +p+K\sin\theta ,\;p+K\sin\theta \right)}^{t}$$

where $y={\left(\theta ,p\right)}^{t}\in {\left[-\pi ,\pi \right]}^{2}$.

The Jacobian matrix of the standard map $f_{K}$ is calculated as follows:

(62) $$Df_{K}\left(y\right)=\left(\begin{array}{cc} 1+K\cos\theta & 1\\ K\cos\theta & 1 \end{array}\right).$$

Thus, $Df_{K}\left(y\right)$ depends on θ and the parameter *K*. Moreover, the dynamics associated with standard map $f_{K}$ are conservative because $|detDf_{K}\left(y\right)|=1$.

Now, consider orbit $\left\{y_{n}\right\}$ associated with the standard map $f_{K}$ such that:

$$y_{n+1}=f_{K}\left(y_{n}\right),\;n=0,1,2,\ldots ,\;y_{0}={\left(1.5,2.0\right)}^{t}.$$

Let $f_{K}^{m}$ be the transformation from $dv_{0}$ to $dv_{m}$ in $R^{2}$. By using the chain rule and $detDf_{K}\left(x\right)=1$ for the standard map $f_{K}$, the following is obtained:

(63) $$dv_{m}=detDf_{K}^{m}\left(v_{0}\right)dv_{0}=dv_{0}.$$

Thus,

(64) $$\lambda_{1}\left(f_{K}\right)+\lambda_{2}\left(f_{K}\right)=\lim_{m\to \infty }\frac{1}{m}\log\left|\frac{dv_{m}}{dv_{o}}\right|=0.$$

As mentioned in [16], the standard map $f_{k}$ is reversible [22]. Thus, LEs $\lambda_{1}\left(f_{K}\right)$ and $\lambda_{2}\left(f_{K}\right)$ of $f_{K}$ satisfy the condition such that $\lambda_{1}\left(f_{K}\right)=-\lambda_{2}\left(f_{K}\right)>0$ according to Theorem 3.2 in [23].

**Figure 15:**
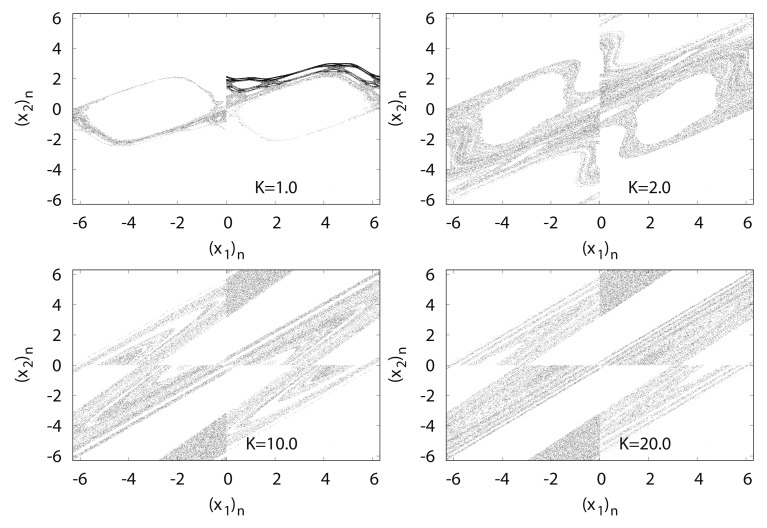
${\left(x_{2}\right)}_{n}$ versus ${\left(x_{1}\right)}_{n}$ for standard map $f_{K}$.

Figure 15 shows typical orbits of the standard map $f_{K}$ with the initial point $\left(\theta_{0},p_{0}\right)=\left(1.5,2.0\right)$. The standard map is composed of the Poincaré’s surface in the kicked rotator section, and $f_{K}$ has a linear structure at approximately K=0. However, with the increase in *K*, the map generates a non-linear structure with chaos under appropriate initial conditions.

**Figure 16:**
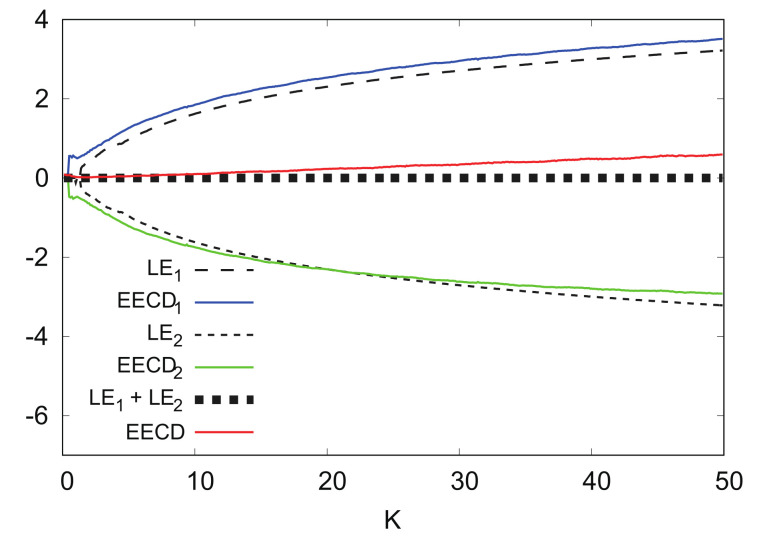
${LE}_{k}$, ${EECD}_{k}$(k=1,2), ${LE}_{1}+{LE}_{2}$ and EECD versus *K* for standard map $f_{K}$.

Figure 16 shows the numerical computation results for ${LE}_{k}$, ${EECD}_{k}$(k=1,2), ${LE}_{1}+{LE}_{2}$, and EECD for the standard map $f_{K}$. Comparisons of ${EECD}_{1}$ and ${LE}_{1}$ indicate that ${EECD}_{1}$ appears larger than the ${LE}_{1}$ in 0≤K≤50 for a certain small value.

Now, consider increasing the number $L^{2}$ of all components of the equipartition of *I*. In principle, downsizing $A_{i}$ by increasing *L*, is necessary to compute ${EECD}_{1}$ more precisely for any *K*, because the Jacobian matrix $Df_{K}\left(y\right)$ of the standard map $f_{K}$ depends on y as well as *K*.

**Figure 17:**
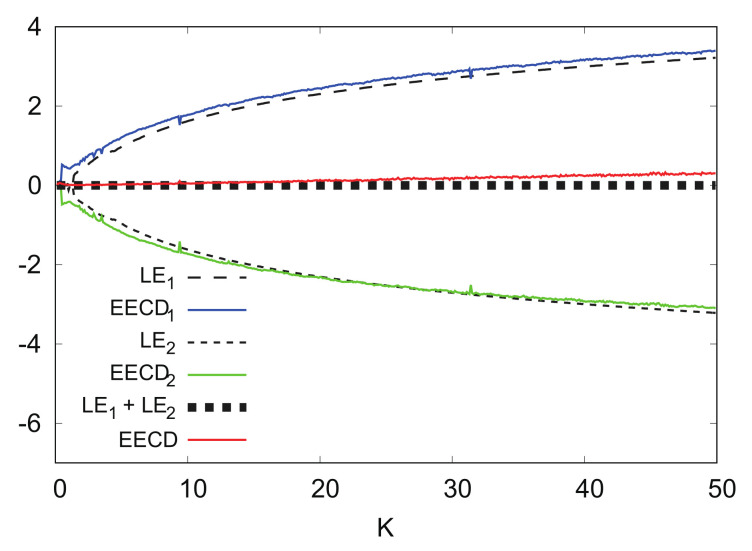
${LE}_{k}$, ${EECD}_{k}$(k=1,2), ${LE}_{1}+{LE}_{2}$, and EECD versus *K* for standard map $f_{K}$ (L=2000).

Figure 17 shows the numerical computation results for ${LE}_{k}$, ${EECD}_{k}$(k=1,2), ${LE}_{1}+{LE}_{2}$, and EECD for the standard map $f_{K}$ at L=2000 instead of L=1000. By increasing *L*, it is possible to reduce the difference between the ${\mathrm{EECD}}_{1}$ and ${\mathrm{LE}}_{1}$ such that the EECD may approach ${\mathrm{LE}}_{1}+{\mathrm{LE}}_{2}$.

## 6. Conclusions

In this study, by reviewing the derivation process of the improved calculation formula of the EECD, it is shown that all the LEs for an aperiodic orbit could be estimated when calculating the EECD; furthermore, a computational algorithm for the EECD is proposed. This computational algorithm is applied to typical one and two-dimensional chaotic maps.

First, the computational algorithm of the EECD is applied to typical one-dimensional chaotic maps, such as the generalized Bernoulli shift and logistic maps. The numerical computation results for these one-dimensional chaotic maps indicate that the EECD is approximately the same as the LE in all chaotic cases.

Thereafter, the computational algorithm of the EECD is applied to two-dimensional typical chaotic maps, such as the generalized baker’s, Tinkerbell, Ikeda, Hénon, and standard maps. The numerical computation results for these typical two-dimensional chaotic maps show that the EECD is approximately the same as the total sum of the LEs in most chaotic cases; however, the *k*th item of the EECD is also approximately the *k*th LE for k=1,2, which can be slightly larger or smaller.

Therefore, it can be concluded that the EECD may be an alternative to the LE for both one and two-dimensional chaotic dynamics. In future studies, attempts will be made to characterize higher-dimensional chaotic dynamics and non-linear real-time series using the EECD. 

## Data Availability

Data sharing not applicable.

## References

[1] Aligood K.T., Sauer T.D., York J.A. (1997). Chaos-An Introduction to Dynamical Systems.

[2] Eckmann J.P., Kamphorst S.O., Ruelle D., Ciliberto S. (1986). Lyapunov exponents from time series. Phys. Rev. A.

[3] Rosenstein M.T., Collins J.J., De Luca C.J. (1993). A practical method for calculating largest Lyapunov exponents from small data sets. Phys. D.

[4] Sato S., Sano M., Sawada Y. (1987). Practical methods of measuring the generalized dimension and the largest Lyapunov exponent in high dimensional chaotic systems. Prog. Theor. Phys..

[5] Sano M., Sawada Y. (1995). Measurement of the Lyapunov spectrum from a chaotic time series. Phys. Let. A.

[6] Wright J. (1984). Method for calculating a Lyapunov exponent. Phys. Rev. A.

[7] Wolf A., Swift J.B., Swinney H.L., Vastano J.A. (1985). Determining Lyapunov exponents from a time series. Physica.

[8] Ohya M. (1998). Complexities and their applications to characterization of chaos. Int. J. Theo. Phys..

[9] Inoue K., Ohya M., Sato K. (2000). Application of chaos degree to some dynamical systems. Chaos Solitons Fractals.

[10] Inoue K., Ohya M., Volovich I. (2002). Semiclassical properties and chaos degree for the quantum baker’s map. J. Math. Phys..

[11] Inoue K., Ohya M., Volovich I. (2009). On a combined quantum baker’s map and its characterization by entropic chaos degree. Open Syst. Inf. Dyn..

[12] Inoue K. (2013). Basic properties of entropic chaos degree in classical systems. Information.

[13] Mao T., Okutomi H., Umeno K. (2019). Investigation of the difference between chaos degree and Lyapunov exponent for asymmetric tent maps. JSIAM Lett..

[14] Mao T., Okutomi H., Umeno K. (2019). Proposal of improved chaos degree based on interpretation of the difference between chaos degree and Lyapunov exponent. Trans. JSIAM.

[15] Inoue K., Mao T., Okutomi H., Umeno K. (2021). An extension of the entropic chaos degree and its positive effect. Jpn. J. Indust. Appl. Math..

[16] Inoue K. (2021). An improved calculation formula of the extended entropic chaos degree and its application to two-dimensional chaotic maps. Entropy.

[17] Barreira L. (2012). Ergodic Theory, Hyperbolic Dynamics and Dimension Theory.

[18] Willems J.C. (1972). Dissipative dynamical systems part 1: General theory. Arch. Rational Mech. Anal..

[19] Ikeda K. (1979). Multiple-valued stationary state and its instability of the transmitted light by a ring cavity system. Opt. Commun..

[20] Ikeda K., Daido H., Akimoto O. (1980). Optical Turbulence: Chaotic Behavior of Transmitted Light from a Ring Cavity. Phys. Rev. Lett..

[21] Wang Q., Oksasoglu A. (2008). Rank one chaos: Theory and applications. Int. J. Bifurc. Chaos..

[22] Roberts J.A.G., Quispel G.R.W. (1992). Chaos and time-reversal symmetry. Order and chaos in reversible dynamical systems. Phys. Rep..

[23] Bessa M., Carvalho M., Rodrigues A. (2015). Generic area-preserving reversible diffeomorphisms. Nonlinearity.

